# Identification and characterization of alternative sites and molecular probes for SARS-CoV-2 target proteins

**DOI:** 10.3389/fchem.2022.1017394

**Published:** 2022-10-31

**Authors:** Suhasini M. Iyengar, Kelly K. Barnsley, Hoang Yen Vu, Ian Jef A. Bongalonta, Alyssa S. Herrod, Jasmine A. Scott, Mary Jo Ondrechen

**Affiliations:** Department of Chemistry and Chemical Biology, Northeastern University, Boston, MA, United States

**Keywords:** SARS-CoV-2, main protease, RNA methyltransferase, Nucleocapsid, POOL, coupled amino acids, secondary sites

## Abstract

Three protein targets from SARS-CoV-2, the viral pathogen that causes COVID-19, are studied: the main protease, the 2′-O-RNA methyltransferase, and the nucleocapsid (N) protein. For the main protease, the nucleophilicity of the catalytic cysteine C145 is enabled by coupling to three histidine residues, H163 and H164 and catalytic dyad partner H41. These electrostatic couplings enable significant population of the deprotonated state of C145. For the RNA methyltransferase, the catalytic lysine K6968 that serves as a Brønsted base has significant population of its deprotonated state *via* strong coupling with K6844 and Y6845. For the main protease, Partial Order Optimum Likelihood (POOL) predicts two clusters of biochemically active residues; one includes the catalytic H41 and C145 and neighboring residues. The other surrounds a second pocket adjacent to the catalytic site and includes S1 residues F140, L141, H163, E166, and H172 and also S2 residue D187. This secondary recognition site could serve as an alternative target for the design of molecular probes. From *in silico* screening of library compounds, ligands with predicted affinity for the secondary site are reported. For the NSP16-NSP10 complex that comprises the RNA methyltransferase, three different sites are predicted. One is the catalytic core at the conserved K-D-K-E motif that includes catalytic residues D6928, K6968, and E7001 plus K6844. The second site surrounds the catalytic core and consists of Y6845, C6849, I6866, H6867, F6868, V6894, D6895, D6897, I6926, S6927, Y6930, and K6935. The third is located at the heterodimer interface. Ligands predicted to have high affinity for the first or second sites are reported. Three sites are also predicted for the nucleocapsid protein. This work uncovers key interactions that contribute to the function of the three viral proteins and also suggests alternative sites for ligand design.

## Introduction

Severe acute respiratory syndrome coronavirus 2 (SARS-CoV-2), the pathogen that causes the COVID-19 global pandemic (Wu F. et al, 2020; Zhou et al., 2020), has currently led to more than 620 million confirmed cases and more than six million deaths in over 200 countries according to the World Health Organization (https://covid19.who.int/). Oral antiviral drugs that act directly on the target of interest are a foundation for the treatment of viral diseases and two oral antiviral medications, Paxlovid and Lagevrio, have received emergency use authorization from the United States Food and Drug Administration (FDA) for the treatment of COVID-19. Given the heavy toll that COVID-19 has taken on human lives and health, as well as the serious social and economic impacts, we must learn as much as possible to characterize the viral components and how they function. Because a wider array of treatments is desired, further characterization of individual viral protein targets is required to develop future chemical probes and high-affinity ligands for COVID-19 and other potential related coronavirus infections.

The SARS-CoV-2 virus is a beta-coronavirus closely related to SARS-CoV and MERS-CoV. SARS-CoV-2 is a positive-strand RNA virus with a single-stranded RNA genome that consists of ∼29800 bases which encodes up to 14 open reading frames (ORFs) (Wu A. et al, 2020). The viral genome encodes four structural proteins, Spike (S), envelope (E), membrane (M) and nucleocapsid (N), and 16 non-structural (NSP1-NSP16), proteins that are essential for the life cycle of the virus (Snijder et al., 2016; Wu A. et al, 2020). It is critical to understand how the viral proteins function and how their function may be modulated. The current drug discovery efforts primarily target the main protease, also called the 3CL-protease (MPro, NSP5, 3CL-Pro), the RNA-dependent RNA polymerase, and the Spike protein (Ramajayam et al., 2011; Wrapp et al., 2020). In this study, the targets of interest are the main protease (MPro), the RNA methyltransferase (MTase, NSP16) and the nucleocapsid protein (N protein). A greater understanding of the function of viral proteins can add to the knowledge of the viral life cycle at the atomic and molecular level. The results of this study can help to understand the function of these viral proteins and to guide strategies for the accelerated development of interventions to mitigate COVID-19.

MPro is one of two internally encoded proteases that hydrolyze the polyproteins at specific locations. Because MPro is essential for viral replication it is a validated drug target for SARS-CoV-2 (Gao et al., 2021; Cao et al., 2022; Huff et al., 2022). The MPro is made up of three domains: domains I (residues 10–99) and II (residues 100–182) have an antiparallel β-barrel structure. Domain III (residues 198–303) has a cluster of helices (Jin et al., 2020; Zhang et al., 2020). During the first step of the hydrolysis reaction, C145 acts as a nucleophile, assisted by H41 that acts as a base catalyst. There are also several binding sites in the catalytic machinery, with the S1 site defining the enzyme’s affinity for glutamine at the P1 position of the peptide substrate. Domain III is involved in MPro dimerization, with the homodimer being proposed to be the active form of the enzyme (Hilgenfeld, 2014). Serial truncation experiments have also demonstrated that the last C-terminal helix in domain III is critical for dimerization (Hsu et al., 2005).

Capping of the viral RNA is critical for the survival and further replication of the virus in cells. For SARS-CoV-2, the 2′-O-methyltransferase (NSP16), with its partner protein NSP10, catalyzes a key step in the capping process. Thus it has been identified as a therapeutic target (Ahmed-Belkacem et al., 2022; Rowaiye et al., 2022; Sulimov et al., 2022). SARS-CoV-2 NSP16 has twelve strands, seven helices, and five 3_10_ helices, whereas NSP10 has a central antiparallel pair of strands and a helical domain with two zinc fingers. The NSP10 zinc coordinating residues are highly conserved across beta-coronaviruses, underlining the necessity for zinc coordination. The X-ray crystal structures highlight the *S*-adenosyl-L-methionine (SAM) and RNA cap substrate-binding pockets, together with the NSP10/NSP16 interface, as potential therapeutic targets. The NSP16 protein catalyzes the transfer of the methyl group from SAM to Cap-0, resulting in the reaction products S-adenosyl homocysteine (SAH) and Cap-1. The catalytic site of NSP16 is a highly conserved motif among class I MTases (K-D-K-E) and contains the residues K6839, D6928, K6968, and E7001 (Bollati et al., 2009; Chen et al., 2011; Decroly et al., 2011). Nsp10-derived peptide inhibitors have been identified as attractive therapeutic targets because they inhibit 2′-O-methyltransferase activity and impair viral replication (Ke et al., 2012; Wang et al., 2015; Ahmed-Belkacem et al., 2022; Sulimov et al., 2022). This 2′-O methyltransferase (MTase) has been shown to be required for coronavirus replication in cell cultures (Decroly et al., 2008; Daffis et al., 2010). NSP10 is an important cofactor for NSP16 and significantly increases NSP16 activity (Sawicki et al., 2005; Minskaia et al., 2006; Decroly et al., 2008; Daffis et al., 2010; Ma et al., 2015; Wang et al., 2015).

The nucleocapsid protein (N protein) is a key component of the viral envelope. The SARS-CoV-2 N protein is a multifunctional RNA-binding protein that is required by the virus for RNA transcription and replication. It plays important roles in the formation of helical ribonucleoproteins during the packaging of the viral RNA genome, controlling viral RNA synthesis in replication/transcription, and modulating infected cell metabolism (Stohlman et al., 1988; Nelson et al., 2000; Cong et al., 2020). Because of its essential roles in the viral lifecycle, the N protein is regarded as a therapeutic target (Wang et al., 2022). A conserved architecture with a β-sheet core of five antiparallel β-sheets, an extended β3-4 hairpin, and an acidic loop with a 3_10_ helix is revealed in the structures of the SARS-CoV-2 N protein RNA binding domain. The primary functions of N protein are to bind to the viral RNA genome and pack it into a long helical nucleocapsid structure or ribonucleoprotein (RNP) complex (Masters & Sturman, 1990; McBride et al., 2014). The conservation of the N protein sequence across coronaviruses and its high immunogenicity make the N Protein an attractive therapeutic target for testing *in silico* (Luo et al., 2006; Yu et al., 2006; Peng et al., 2008; Tatar et al., 2021).

We report herein on the computationally predicted binding sites, on interactions between these sites, and on library compounds that possibly bind to these sites, for three SARS-CoV-2 protein targets: the main protease (MPro, NSP5, 3CL-Pro), the 2′-O-methyltransferase (MTase, NSP16), and the nucleocapsid protein (N protein).

## Materials and methods

### Protein structure retrieval and preparation

#### Protein preparation steps for structures retrieved from the PDB

Recently deposited structures of the SARS-CoV-2 MPro, MTase and N protein were obtained from the protein data bank (PDB) (Table 1). A Protein Reliability Report using Maestro was created with a structure analysis panel to compare the reliability of the structures (Supplementary Figure S1). Before running POOL (Tong et al., 2009; Somarowthu et al., 2011; Somarowthu & Ondrechen, 2012) on these structures, they were prepared and analyzed in YASARA (Krieger & Vriend, 2014). Each structure was loaded into a new YASARA workspace from the PDB, then cleaned to add any missing atoms. Water molecules, cofactors, and ligands were deleted. A simulation cell extending 5.0 Å around all atoms was created, solvated with a 0.999% solution of NaCl at pH = 7.0, and then p*K*_a_ prediction and energy minimization were performed using the YAMBER3 force field. The resulting cleaned structures were used as the input structures for POOL. The Protein Preparation Wizard (Sastry et al., 2013) in Maestro was used to further prepare them before docking with Glide (Halgren et al., 2004). The final step is restricted minimization, which provides controls for optimizing the corrected structure, easing any strain, and fine-tuning the placement of functional groups.

**TABLE 1:** Selected SARS-COV-2 protein structures retrieved from the PDB.

PDB Code	Structure	Resolution (Å)	Reference
6LU7	MPro in complex with an inhibitor N3	2.16	Jin et al. (2020)
6W4H	NSP16-NSP10 Complex	1.80	Rosas-Lemus et al. (2020)
7DE1	Nucleocapsid protein C-terminal RNA binding domain	2.00	Yang et al. (2021)

#### Protein preparation steps for homology models

**FIGURE 1:**
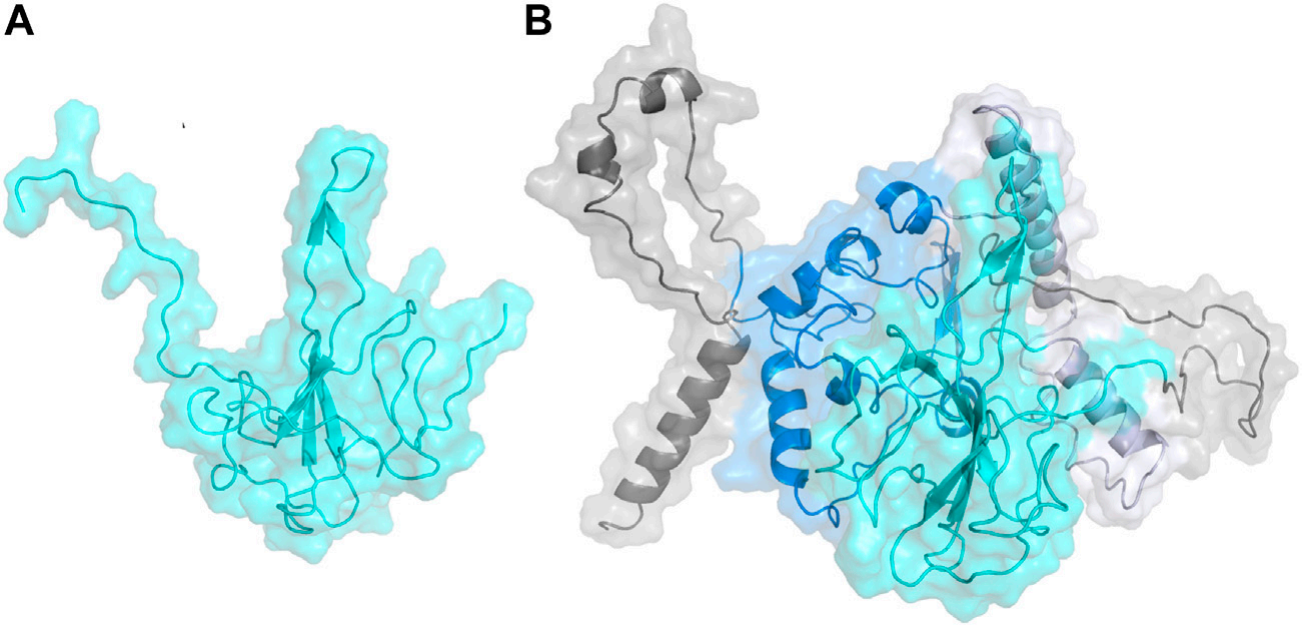
**(A)** Homology model built on YASARA for N-terminal domain of the Nucleocapsid protein shown in cyan. **(B)** Homology model built on I-TASSER for the full length Nucleocapsid protein with domains colored as: N-terminal domain—cyan; C-terminal domain—blue; linker region—lavender; other regions—gray.

The N Protein N-terminal RNA binding domain model structure was built with the homology model module in YASARA (Krieger et al., 2009) using a series of templates from the PDB. These structures were obtained from a BLAST search of the N Protein sequence (UniProtKB accession #P59595) (https://covid-19.uniprot.org/uniprotkb/P59595#Names%20&%20Taxonomy) on the PDB sequence database. The model was built from selected template structures with sequence homology to N Protein: SARS-CoV-2 nucleocapsid protein N-terminal binding domain (PDB ID:6M3M) (Kang et al., 2020); RNA binding domain of nucleocapsid phosphoprotein from SARS-CoV-2 (PDB ID: 6VYO) (https://www.rcsb.org/structure/6VYO); C-terminal dimerization domain of Nucleocapsid Phosphoprotein from SARS-CoV-2 (PDBID: 6WJI) (https://www.rcsb.org/structure/6WJI); and the N-Terminal binding domain of the SARS-CoV-2 nucleocapsid phosphoprotein (PDBID: 6YI3) (Dinesh et al., 2020). Using these four structures as templates, a hybrid model for the N-terminal RNA binding domain of N protein was built in YASARA. Figure 1A shows the hybrid model generated for SARS-CoV-2 N Protein. In addition, a full-length model consisting of the entire N Protein structure was also built using the I-TASSER Server (Yang et al., 2015). Figure 1B shows the full-length model generated for SARS-CoV-2 N Protein. Both these N Protein models were further validated using structure evaluation servers, including ERRAT (Colovos & Yeates, 1993), VERIFY-3D (Eisenberg et al., 1997), the servers in the Structural Analysis and Verification Server (SAVES) (Lüthy et al., 1992), and QMean (Benkert et al., 2011) (Supplementary Figure S2). Then, the model structures were prepared as described above before running POOL (Tong et al., 2009; Somarowthu et al., 2011; Somarowthu & Ondrechen, 2012) and docking.

### Ligand database retrieval and preparation

The ligands were obtained from the following databases: a) ZINC FDA library (https://zinc15.docking.org/substances/subsets/fda/) b) CAS Antiviral set (https://www.cas.org/covid-19-antiviral-compounds-dataset) c) Enamine FDA library (https://enamine.net/hit-finding/compound-collections/bioreference-compounds/fda-approved-drugs-collection) and d) Antiviral library consisting of compounds from: Selleck Chemicals Antiviral Library, Enamine Antiviral Library, and Asinex Antiviral Library. The ligands were prepared using the LigPrep (Schrödinger Release 2020–2: LigPrep, Schrödinger, LLC, New York, NY, 2021) tool.

### Binding Site detection

**FIGURE 2:**
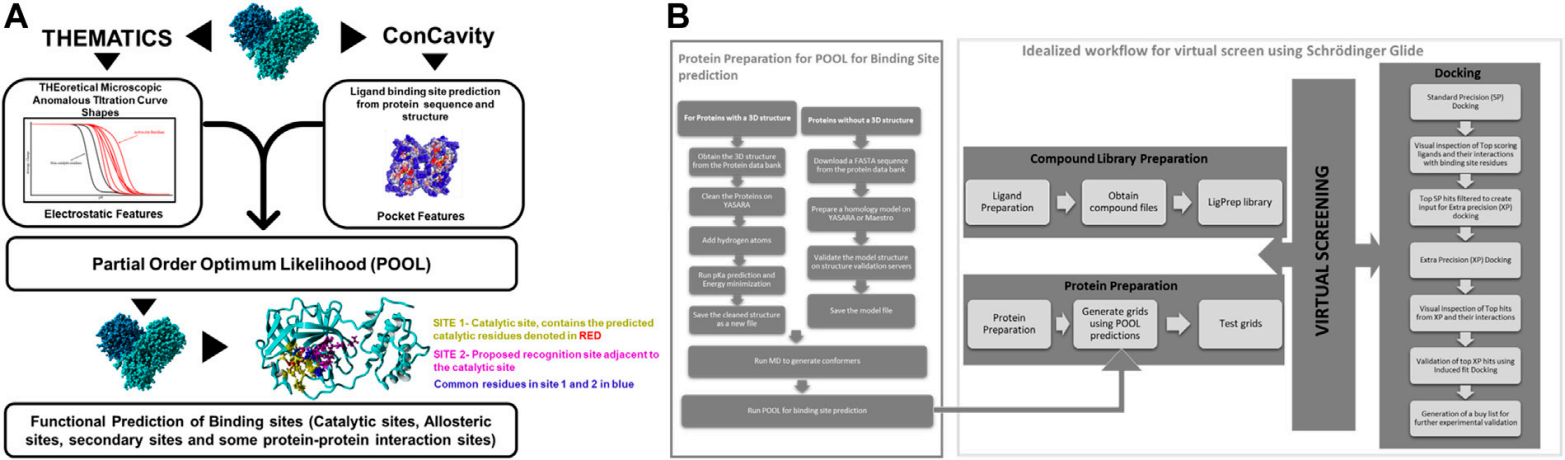
**(A)** A schematic representation of Partial Order Optimum Likelihood (POOL), our machine learning method to predict biochemically active sites. **(B)** Virtual screening workflow adopted in this project in conjunction with POOL.

For the binding site prediction, Partial Order Optimum Likelihood (POOL) (Tong et al., 2009; Somarowthu et al., 2011; Somarowthu and Ondrechen, 2012) was used (Figure 2A). POOL is a machine learning method that predicts biochemically active sites, including catalytic sites, allosteric sites, and exosites, some of which may not be detected by other predictive methods, from the 3D structure. POOL generates a rank-ordered list of all the amino acids in the protein structure in the order of likelihood of biochemical activity and the top 10% of the rank-ordered list are further visualized to predict the binding site pockets. The input features for POOL consist of electrostatic properties of the local environment (Ko et al., 2005; Wei et al., 2007; Tong et al., 2009; Somarowthu et al., 2011) and surface topological metrics from the structure-only version of ConCavity (Capra et al., 2009).

### Virtual screening and analysis

Molecular Docking was performed using Schrödinger Glide (Friesner et al., 2004). For docking in Schrödinger Glide, the protein was minimized and optimized using the Protein Preparation Wizard and the grid for docking was prepared using Receptor Grid Generation using clusters of residues from the top 10% of the POOL predicted residues as the centroid for ligand placement in Schrödinger 2020–3. Molecular Docking was performed on the Discovery Cluster at the Massachusetts Green High-Performance Computing Center using Glide. Glide Standard Precision (SP) (Halgren et al., 2004) was used as an initial screen and top predicted ligands with docking score of <=-7 kcal/mol were then docked with Glide Extra Precision (XP) (Friesner et al., 2006). The top hits from Glide XP were further used as input for the Induced Fit Docking (Sherman et al., 2006a; Sherman et al., 2006b; Farid et al., 2006) method in some cases on Schrödinger to account for the conformational flexibility of the protein and the ligand at the docking site. The top hits from each XP simulation were used to perform postprocessing using Schrodinger’s Virtual Screening Workflow and Prime molecular mechanics/generalized Born surface area (MM- GBSA) calculations using the default settings. The protein-ligand complexes were then ranked on the basis of their binding free energy calculations. The workflow of our strategic modeling and *in-silico* screening is shown in detail in Figure 2B.

### Residue interaction analysis

Pairwise Coulomb potential energies of interaction between amino acid side chains, and the free energies of desolvation of the amino acid side chains, were calculated by a linear Poisson-Boltzmann method (Antosiewicz et al., 1994; Baker et al., 2001; Shen et al., 2003; Dolinsky et al., 2004). The intrinsic p*K*_a_s were calculated from the desolvation energy as (Ullmann, 2003; Coulther et al., 2021; Iyengar et al., 2022):

$$pK_{a}\left(intrinsic\right)=pK_{a}\left(\mathrm{mod}el\right)-\gamma \cdot \Delta \Delta G/\left[\ln\left(10\right)RT\right]$$ (1)

where the intrinsic p*K*_a_ is the p*K*_a_ of an amino acid side chain in the hypothetical protein structure where all other ionizable groups are in their electrically neutral state. The model p*K*_a_ is the p*K*_a_ of the side chain of the free amino acid in solution. For cation-forming side chains, γ = +1; γ = -1 for anion-forming side chains. ΔΔG is the Gibbs free energy of desolvation of the side chain in the hypothetical neutral protein structure, relative to the free amino acid in aqueous solution.

## Results and discussion

### Main protease (MPro, 3CL-Pro or NSP5)

#### POOL predicts a secondary recognition site for the main protease

The SARS-CoV-2 MPro structure consists of three domains: domain I has residues 10–99; domain II residues 100–182, and domain III residues 198–303. The active site is located on a cleft between domains I and II and includes a H41- C145 catalytic dyad. The major subsites in the MPro active site, where the substrate binds, have been identified (Jin et al., 2020). F140, L141, N142, H163, E166, and H172 make up the S1 subsite. A small portion of S1 subsite is further separated by N142 making up the S1’ subsite, which consists of Y25, Y26, and L27, whereas H41, M49, Y54, M165, and D187 make up the hydrophobic S2 subsite. M165, L167, F185, Q189, and Q192 amino acids make up the S4 binding subsite.

**FIGURE 3:**
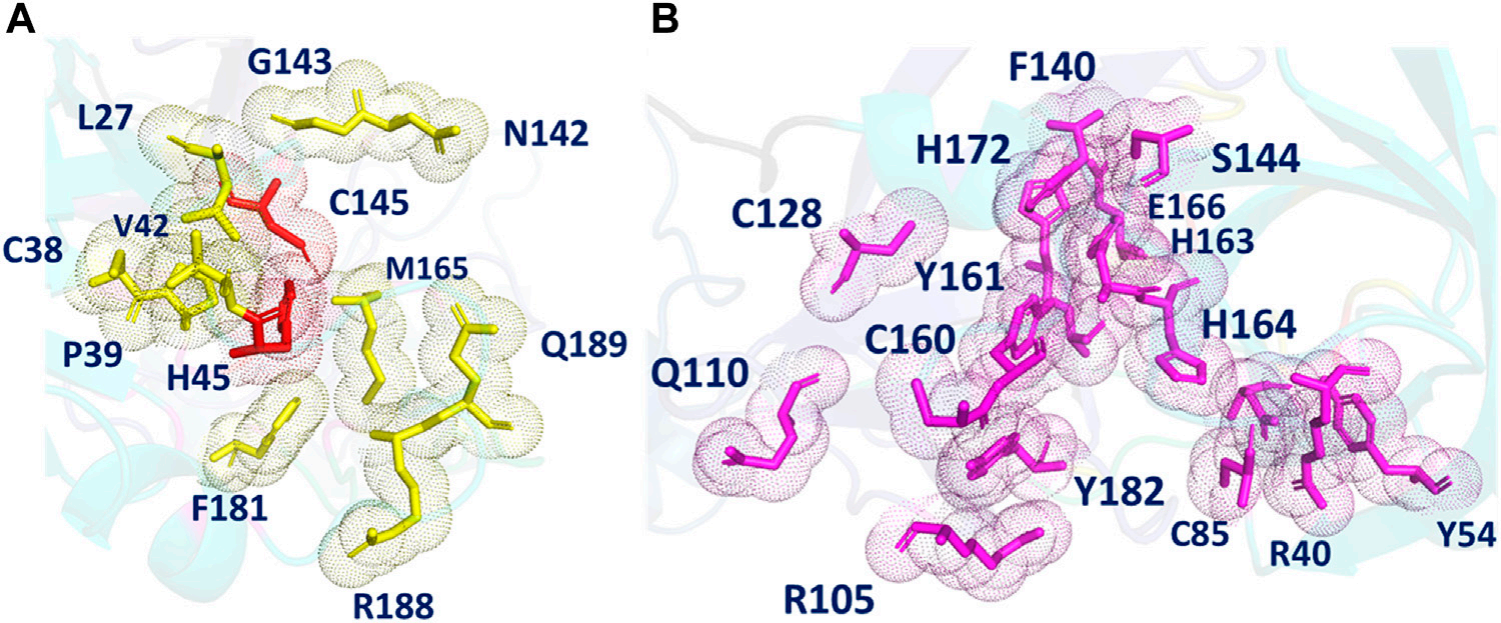
**(A)** The different POOL-predicted pockets for the SARS-CoV-2 Main Protease (PDB ID: 6LU7). The POOL predicted residues in pocket one shown in yellow include the catalytic dyad H41-C145, shown in red. **(B)** The POOL-predicted secondary recognition pocket, shown in magenta, are the residues surrounding the catalytic dyad.

The POOL-predicted residues for the Main Protease monomer (PDB ID: 6LU7, Figure 3) form two clusters near the site of proteolysis. One surrounds a pocket containing the catalytic site and consists of: L27, C38, P39, H41, V42, N142, G143, C145, M165, F181, R188, and Q189, including the two catalytic residues H41 and C145. This cluster also includes N142, L27, and M165, previously labeled as S1, S2, and S4, respectively. POOL predicts a second cluster that surrounds a pocket adjacent to the catalytic site and consists of: R40, Y54, C85, R105, Q110, C128, F140, L141, S144, C160, Y161, H163, H164, E166, H172, A173, Y182, and D187. This secondary recognition site includes S1 residues from Domain II, F140, L141, H163, E166, and H172. It also includes D187, previously identified as a member of S2.

#### Recognition peptide sequence docked on the main protease interacts with the POOL predicted residues

**FIGURE 4:**
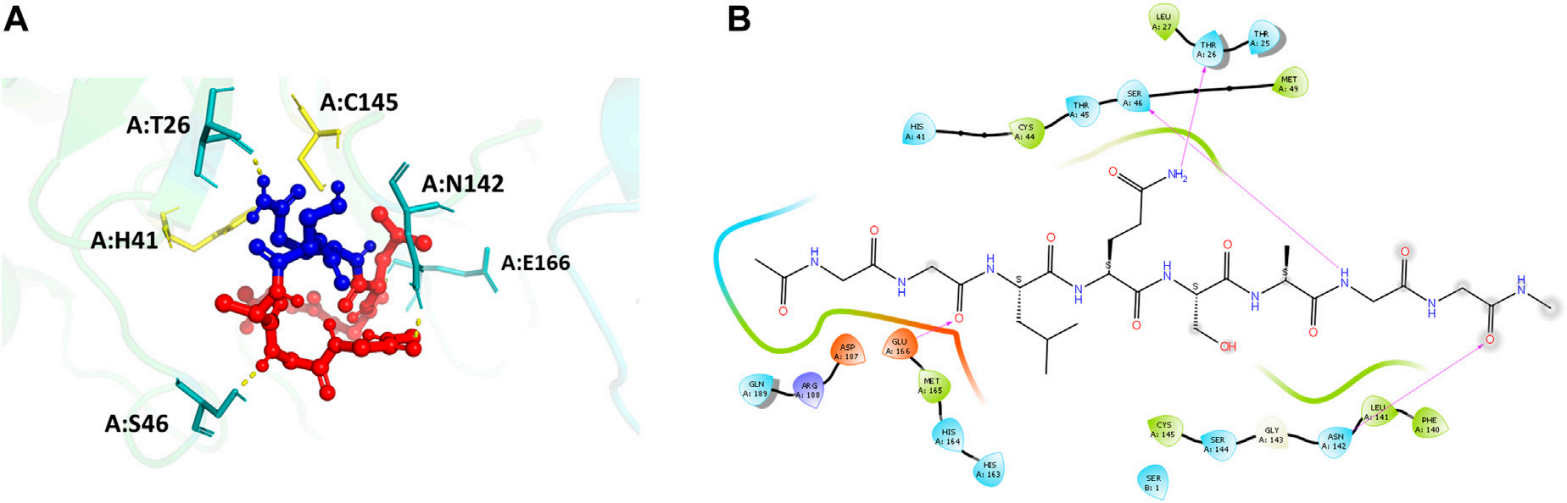
**(A)** Docking pose of the recognition peptide LQ↓SAG with two glycine caps (GG-LQSAG-GG) docked at the active site (in yellow) of the SARS-CoV-2 MPro with chain A in green and chain B in cyan. The peptide GG-LQSAG-GG is shown as red balls and sticks with the cleavage sequence Q↓S shown in blue. The H-bond interactions are shown in yellow and the interacting residues in teal. **(B)** The ligand interaction diagram showing the docked pose of GG-LQSAG-GG and the residues forming a pocket around it.

The main protease recognition sequence is LQ↓SAG. To understand the how the polypeptide sequence interacts with the residues around the active site of the main protease, a peptide fragment, capped on the N- and C- terminal sides with two glycine residues, GG-LQSAG-GG was docked into the protease dimer structure using the Schrodinger Peptide Docking algorithm. The peptide was docked at the catalytic site to identify its interactions with the amino acids surrounding the active site pocket. The docking scores of the complex with the MPro receptor are represented in Table 2. The peptide GG-LQSAG-GG binds to the catalytic pocket with the recognition sequence approaching the two catalytic residues H41-C145, as seen in Figure 4. It has a good docking score and interactions with some of the residues within the S1, S1', S2, and S4 subsites including the active site residues H41 and C145. The peptide GG-LQSAG-GG (Figures 4A,B) gives a docking score of -9.15 kcal/mol and MM-GBSA Binding energy of -31.04 kcal/mol. The GG-LQSAG-GG peptide lies in a pocket (A chain) formed by F140, L141, N142, H163 and E166 which belong to the S1 subsite, T25, T26, and L27 which belong to the S1’ subsite, H41, M49 and D187 from the hydrophobic S2 subsite and M165, and Q189 belonging to the hydrophobic S4 subsite. Some other residues forming a pocket around the peptide are C44, T45, S46, H164, G143, S144, C145, and R188 from the A Chain and Ser1 from the B Chain. Four hydrogen bonds are found between the peptide and T26, S46, N142, and E166, from the A Chain (Figures 4A,B).

**TABLE 2:** Peptide Docking scores with the SARS-CoV-2 Main Protease.

Peptide name	Sequence	Docking score (kcal/mol)	MM-GBSA score (kcal/mol)
GG-LSQAG-GG	GGLQSAGGG	-9.15	-31.0

#### Key interactions with the catalytic residues for MPro

Key interactions that facilitate the catalytic activity of H41 and C145 were analyzed. C145, in order to serve as a nucleophile, must have significant population of the deprotonated state of its side chain. This is achieved through coupling to three nearby histidine residues, the dyad partner H41, the S1 residue H163, and H164, as shown in Table 3. The strong electrostatic coupling between C145 and these three histidines, with the intrinsic p*K*_a_ of the anion-forming residue higher than that of each of the cation-forming residues, leads to an expanded buffer range and significant populations of both protonation states that is necessary for catalysis (Koumanov et al., 2002; Ringe et al., 2004; Coulther et al., 2021; Iyengar et al., 2022). Indeed, significant population of the deprotonated state of C145 has been implied in a recent report suggesting covalent binding of selected ligands by C145 (Mohapatra et al., 2021). Similarly, the catalytic H41 must have significant population of both protonation states to exchange a proton with the thioester intermediate. This is achieved through the coupling to C145, and also to H164, wherein the two like-charged histidine side chains are strongly coupled to each other and have matched (<1 pH unit difference) intrinsic p*K*_a_s (Table 3) (Koumanov et al., 2002; Coulther et al., 2021; Iyengar et al., 2022).

**TABLE 3:** Computed pairwise energies of electrostatic interaction (kcal/mol) between the two catalytic residues C145 and H41 and their strongest coupling partners in the SARS-CoV-2 main protease. Intrinsic p*K*_*a*_s for each residue are also listed.

Top couplers to C145: p*K*_*a*_ (intrinsic) = 8.9			Top couplers to H41: p*K*_*a*_ (intrinsic) = 5.6		
Residue	|E|(kcal/mol)	p*K*_*a*_ (intrinsic)	Residue	|E|(kcal/mol)	p*K*_*a*_ (intrinsic)
H41	1.3	5.6	Y54	1.7	11.7
H163	1.0	5.0	H164	1.7	5.1
H164	0.94	5.1	C145	1.3	8.9
Y54	0.58	11.7	D187	1.2	5.2
Y161	0.56	11.9	C44	1.0	9.7

#### Potential SARS-CoV-2 main protease inhibitors that bind to the secondary recognition MPro POOL predicted site

Docking reveals potential ligand candidates that bind to the MPro secondary recognition site predicted by POOL; the top five are: 989–51–5 (Epigallocatechin gallate); ZINC000085540219 (Ioxilan); Z1563146136 (Acarbose); ZINC000003914596 (Saquinavir) and ZINC000003830947 (Iopamidol) (Supplementary Table S1). Supplementary Table S1 shows the Glide docking method, docking scores and details of the ligand-protein interactions. The list includes antidiabetic agents and protease inhibitors. The docking score ranges for Induced fit docking on Glide were between -14 and -11 kcal/mol. The interactions between the ligand-protein complex are shown in Supplementary Figure S3 and listed in Supplementary Table S4. All the compounds bind at the POOL predicted secondary site and some of the important H-bond interactions are observed with the residues N142, H164, E166, and Q189; these are similar to the interactions observed with the recognition peptide sequences. Some of the important π- π interactions are observed with H41 and H164.

**FIGURE 5:**
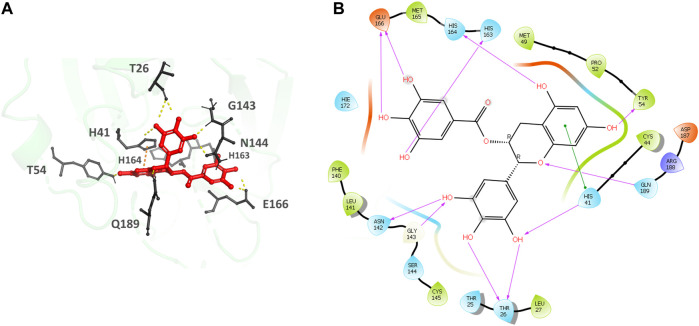
**(A)** Epigallocatechin gallate bound to the monomer of the SARS-CoV-2 MPro at the POOL predicted secondary site. The protein backbone is shown in green cartoon representation, ligand in red with the residues in gray. The hydrogen bonds are shown as yellow dashes and π- π stacking interactions as orange dashes. **(B)** Ligand interaction diagram of Epigallocatechin gallate at the secondary site. The hydrogen bonds are shown as pink arrows and π- π stacking interactions are shown as green lines.

Epigallocatechin gallate (Figures 5A,B), the top hit, binds to the MPro secondary recognition site with an IFD-XP Score of -14.12 kcal/mol. It forms 11 H-bond interactions with the residues T26, H41, Y54, N142, H163, H164, E166, and Q189 and a π- π interaction is observed with H41. The residues forming a pocket around the binding pose of Epigallocatechin gallate are T25, T26, L27, H41, C44, M49, P52, Y54, F140, L141, N142, G143, S144, C145, H163, H164, M165, E166, D187, R188 and Q189.

### Methyltransferase (MTase, NSP16/NSP10 complex)

#### POOL predicts multiple sites for the methyltransferase

The NSP16 crystal structure (PDB ID: 6W4H) is made up of the polyprotein pp1ab residues 6,799 to 7,096. The catalytic core of the NSP16 forms a Rossmann-like beta-sheet fold with seven β-strands and one antiparallel β-strand (β7) which is sandwiched between 11 α-helices and 20 loops. There are three β-strands (β′1, β′2, and β′3) in the NSP10 protein, which have pp1a residues 4,272–4,392, that form a central antiparallel β-sheet at its core. On one side of the β -sheet is a large loop that directly interacts with NSP16 and stabilizes the heterodimer complex. On the other side of this β-sheet, six helices and loops form two zinc finger motifs. It has been reported that coronaviruses use zinc fingers to non-specifically bind RNA (Matthes et al., 2006; Chen et al., 2011). There are two Zn^2+^-binding sites; the first one is coordinated by C4327, C4330, H4336, and C4343 and the second one is coordinated by C4370, C4373, C4381, and C4383. The NSP16 protein catalyzes the transfer of the methyl group from SAM to Cap-0, resulting in the reaction products S-adenosyl homocysteine (SAH) and Cap-1 (Nencka et al., 2022). The adenosine moiety is stabilized by residues F6947, D6912, L6898, C6913, and M6929. The sugar moiety is stabilized by G6871 and D6897 residues, as well as two molecules of water which interact with N6899. The methionine moiety interacts with the residues D6928, Y6845, N6841, and G6871.

**FIGURE 6:**
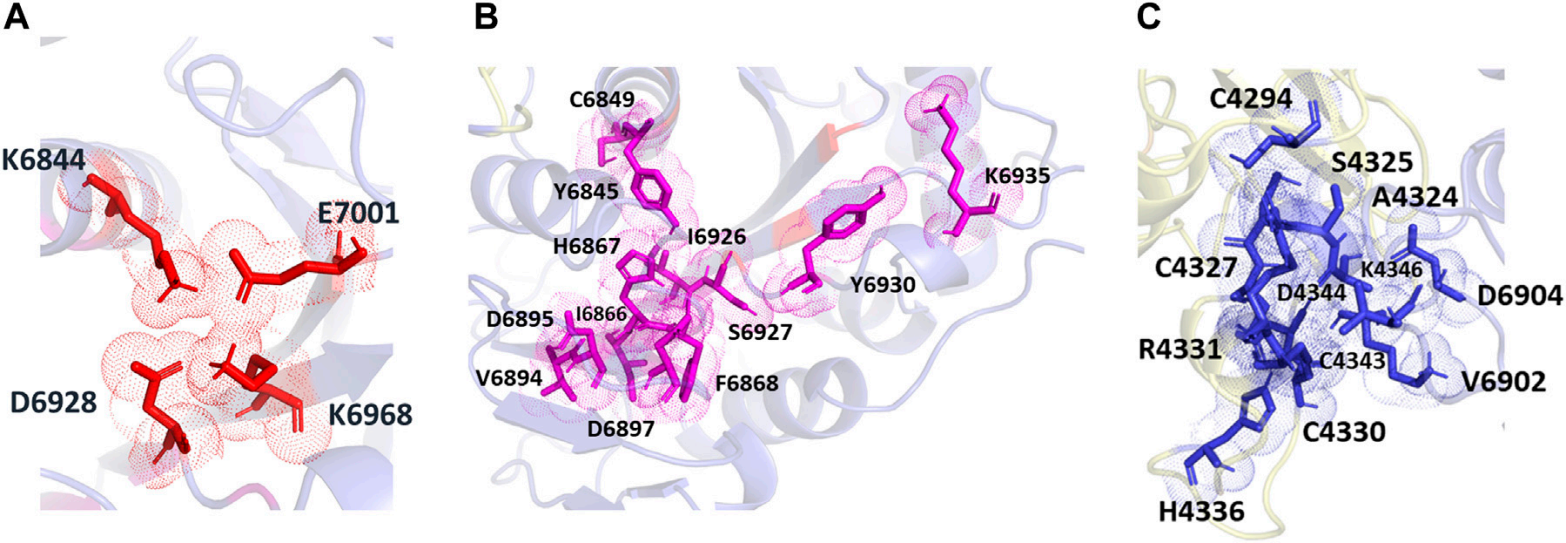
The different POOL predicted sites for the SARS-CoV-2 RNA methyltransferase (NSP16/NSP10 complex) **(A)** The POOL-predicted residues in site 1, which contains the D-K-E part of the K-D-K-E conserved catalytic motif (D6928, K6968, and E7001) with an additional K6844, are shown in red **(B)** POOL-predicted site 2, containing residues surrounding the catalytic motif, is shown in magenta. **(C)** Site 3, and the POOL-predicted residues at the dimer interface, in blue.

The POOL-predicted residues for the methyltransferase form three clusters, as shown in Figure 6. The first one is located at the conserved catalytic K-D-K-E motif found in methyltransferases, and includes the catalytic D6928, K6968, and E7001, with an additional K6844 residue (colored in red). The second cluster surrounds the catalytic pocket and includes Y6845, C6849, I6866, H6867, F6868, V6894, D6895, D6897, I6926, S6927, Y6930, and K6935 (shown in magenta). The third POOL-predicted site for the NSP16-NSP10 complex lies at the heterodimer interface and consists of C4294, A4324, S4325, C4327, C4330, R4331, D4335, H4336, C4343, D4344, K4346, and C4383 of the NSP10 chain and V6902, D6904 of the NSP16 chain (shown in blue).

#### Key interactions with the catalytic residues for the methyltransferase

For each of the catalytic residues in the K-D-K-E motif, the top five couplers with the highest pairwise potential energies of interaction are shown in Table 4. Lysine-6968 serves as the base that enables the 2’ oxygen atom of the RNA to attack the methyl group of SAM (Nencka et al., 2022). The deprotonated state of K6968 is significantly populated, first by the strong electrostatic coupling to K6844, wherein the two lysine residues have closely matched intrinsic p*K*_a_s (Table 4), and second by strong coupling to Y6845 (and to a lesser extent to Y6930), wherein the anion-forming tyrosine has an intrinsic p*K*_a_ higher than that of the lysine (Koumanov et al., 2002; Coulther et al., 2021; Iyengar et al., 2022). Strong coupling to the two anion-forming residues, D6928 and E7001, strengthens the basicity of K6968.

**TABLE 4:** Computed pairwise energies of electrostatic interaction (kcal/mol) between three of the members of the catalytic tetrad D-K-E, D6928, K6968, and K7001, plus the coupled K6844, and their five strongest coupling partners for each, in the SARS-CoV-2 RNA methyltransferase. Intrinsic p*K*_*a*_s for each residue are also listed.

Top couplers to D6928: p*K*_*a*_ (intrinsic) = 4.6			Top couplers to E7001: p*K*_*a*_ (intrinsic) = 5.0			
Residue	|E|(kcal/mol)	p*K*_*a*_ (intrinsic)	Residue	|E|(kcal/mol)	p*K*_*a*_ (intrinsic)
K6968	2.6	9.8	K6844	2.4	9.4
Y6845	2.4	10.7	K6968	2.0	9.8
K6844	1.5	9.4	D6928	1.2	4.6
E7001	1.2	5.0	Y7026	0.90	12.5
H6867	1.0	3.1	Y6845	0.78	10.7
**Top couplers to K6844: p*K***_***a***_ **(intrinsic) = 9.4**			**Top couplers to K6968: p*K***_***a***_ **(intrinsic) = 9.8**		
**Residue**	**|E|(kcal/mol)**	**p*K***_***a***_ **(intrinsic)**	**Residue**	**|E|(kcal/mol)**	**p*K***_***a***_ **(intrinsic)**
E7001	2.4	5.0	D6928	2.6	4.6
K6968	1.8	9.8	E7001	2.0	5.0
D6928	1.5	4.6	K6844	1.8	9.4
Y6845	0.91	10.7	Y6845	0.91	10.7
Y6930	0.53	10.2	Y6930	0.77	10.2

#### Potential SARS-CoV-2 Methyltransferase (NSP16/NSP10 Complex) inhibitors that bind to the MTase POOL-predicted site containing the conserved catalytic motif

To verify the docking method, the known ligand sinefungin was docked into the catalytic site, resulting in a pose similar to that of the reported complex structure (PDB ID 6WKQ) (Rosas-Lemus et al., 2020) with a docking score of -8.2 kcal/mole. Docking studies and analysis yield potential ligand candidates that bind to the MTase conserved catalytic motif. Examples are mostly from the Chemical Abstracts Service (CAS) Antiviral Database and include: CAS ID# 435297–57–7 (1H-1,2,4-Triazole-3-carboxamide, 1-β-D-ribofuranosyl-, 5′-[6-hydrogen (2R)-2-aminohexanedioate); 435297-58-8 (1H-1,2,4-Triazole-3-carboxamide, 1-β-D-ribofuranosyl-, 5′-[6-hydrogen (2S)-2-aminohexanedioate); 1312805-81-4 (Adenosine, 1′-[3-(aminocarbonyl)-1H-1,2,4-triazol-1-yl]-1′-de (6-amino-9H-purin-9-yl)adenylyl-(2′→5′)-1′-[3-(aminocarbonyl)-1H-1,2,4-triazol-1-yl]-1′-de (6-amino-9H-purin-9-yl)adenylyl-(2′→5′)-1′-[3-(aminocarbonyl)-1H-1,2,4-triazol-1-yl]-1′-de (6-amino-9H-purin-9-yl)-(Acl))); 1002334-92-0 (1H-1,2,4-Triazole-3-carboxamide, 1-[5-O-[5-(β-D-galactopyranosyloxy)-1-oxopentyl]-β-D-ribofuranosyl]-(Acl)); and 435297-32-8 (L-Arginine, 5′-ester with 1-β-D-ribofuranosyl-1H-1,2,4-triazole-3-carboxamide- (9Cl)) (Supplementary Table S2). Supplementary Table S2 shows the Glide docking method, docking scores, and MM-GBSA scores, along with the specific ligand-protein interactions. The docking score ranges for Extra Precision (XP) Gscore from Glide were between -14 and -13 kcal/mol. The interactions in the ligand-protein complex are shown in Supplementary Figure S4 and listed in Supplementary Table S2. All the compounds bind at the POOL-predicted site containing the conserved catalytic motif and important H-bond interactions are observed with the residues K6844, G6911, C6913, D6928, and K6968.

**FIGURE 7:**
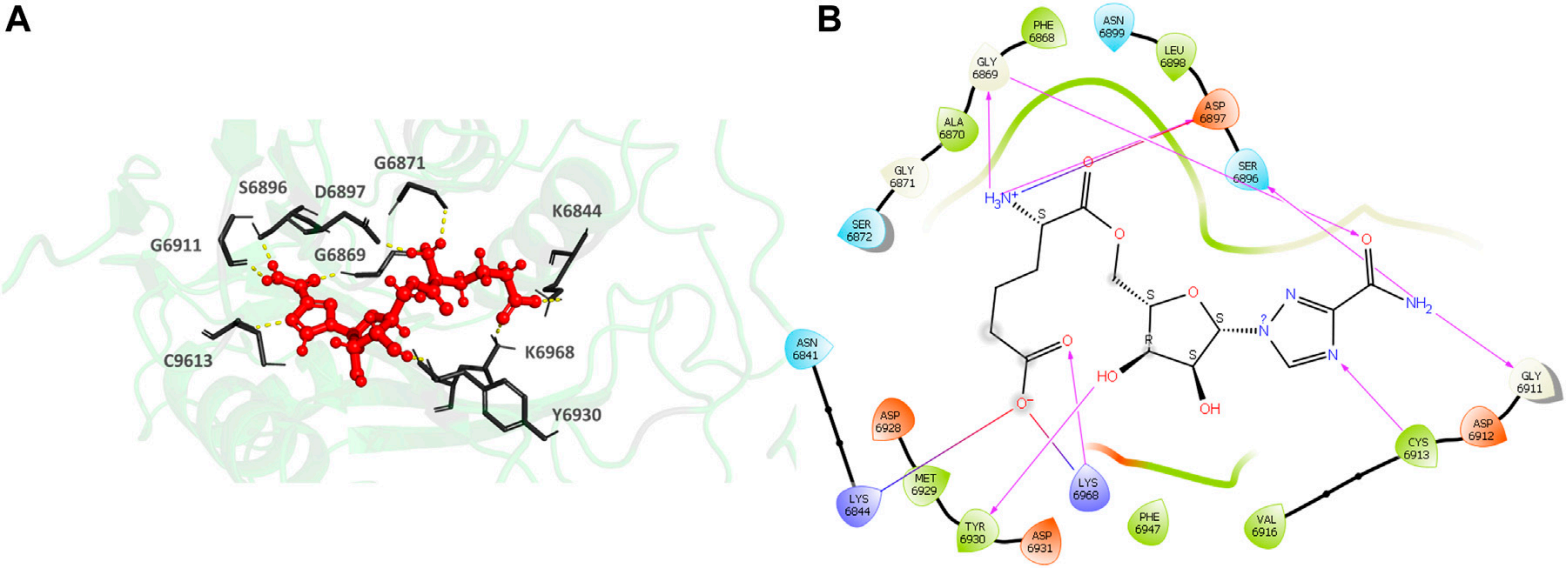
**(A)** CAS#435297–57–7 bound to the SARS-CoV-2 NSP16/NSP10 complex (MTase) at the POOL-predicted site including the conserved catalytic motif. The protein backbone is shown in cartoon representation in green, ligand in red with the residue side chains in gray. The hydrogen bonds are shown as yellow dashes. **(B)** Ligand interaction diagram of CAS#435297–57–7 at the POOL-predicted site containing the conserved catalytic motif. The hydrogen bonds are shown as pink arrows and salt bridges in a bluish-red line.

435297-57-7 (1H-1,2,4-Triazole-3-carboxamide, 1-β-D-ribofuranosyl-, 5′-[6-hydrogen (2R)-2-aminohexanedioate) (Figures 7A,B), the top hit, binds to the MTase conserved catalytic pocket with an XP Gscore of-14.88 kcal/mol. It forms seven H-bonds and interactions with the residues S6896, D6897, G6869, G6911, C6913, Y6930 and K6968. The residues forming a pocket around the binding pose of 435297-57-7 are N6841, K6844, A6870, G6869, F6868, S6872, G6871, G6911, D6912, C6913, V6916, D6928, M6929, Y6930, D6931, F6947, K6968.

#### Potential SARS-CoV-2 Methyltransferase (NSP16/NSP10 Complex) inhibitors that bind to the MTase second POOL-predicted pocket surrounding the conserved catalytic motif

Docking studies and analysis yield potential ligand candidates that bind to the second POOL-predicted NSP16 pocket surrounding the catalytic motif. Examples include: CAS ID# 926902-14-9 (Adenosine, 5′→P-ester with thiotetraphosphoric acid ([(HO) (HS)P(O)OP(O) (SH)]2O), P‴→5′-ester with uridine); 162754-90-7 (β-D-arabino-Adenosine, 5′-O-phosphonoadenylyl-(2′→5′)-adenylyl-(2′→5′)- (9Cl)); 188560-02-3 (Inosine 5′-(pentahydrogen tetraphosphate), P′→5′-ester with inosine); 217807-08-4 (Adenosine, 5′-O-[hydroxy [[hydroxy (phosphonooxy) phosphinyl]oxy] phosphinyl]adenylyl-(2′→5′)-adenylyl-(2′→5′)-1′-[3-(aminocarbonyl)-1H-1,2,4-triazol-1-yl]-1′-de (6-amino-9H-purin-9-yl)- (9Cl)); and 217807-10-8 (Adenosine, 5′-O-[hydroxy (phosphonooxy)phosphinyl]adenylyl-(2′→5′)-1′-[3-(aminocarbonyl)-1H-1,2,4-triazol-1-yl]-1′-de (6-amino-9H-purin-9-yl)adenylyl-(2′→5′)- (9Cl)), (Supplementary Table S3). Supplementary Table S3 shows the Glide docking method, docking scores, MM-GBSA scores and the specific interactions with amino acids. The list consists mainly of antiviral ligands from the Chemical Abstract Service (CAS) Antiviral Database. The docking score ranges for Extra Precision (XP) docking on Glide were between -14 and -13 kcal/mol. The interactions between the ligand-protein complex are shown in Supplementary Figure S5 and listed in Supplementary Table S3. All the compounds bind at the POOL predicted NSP16 pocket surrounding the catalytic motif and some of the important H-Bond interactions are observed with the residues K6844, D6897, D6912, C6913, D6928, Y6930, and K6935. Some of the important π- π interactions are observed with Y6828 and F6947 of the NSP16 chain.

**FIGURE 8:**
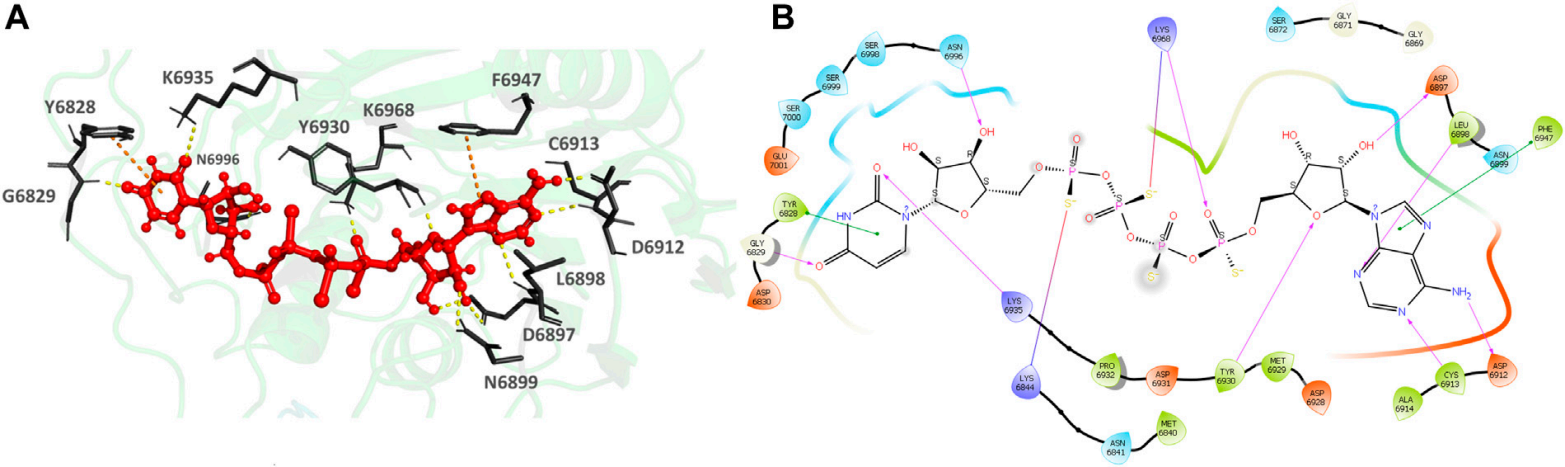
**(A)** CAS# 926902–14–9 bound to SARS-CoV-2 NSP16/NSP10 complex (MTase) at the POOL-predicted residues surrounding the conserved catalytic motif, Site 2. The protein backbone is shown in cartoon representation in green, ligand in red with the residue side chains in gray. The hydrogen bonds are shown as yellow dashes. **(B)** Ligand interaction diagram of CAS# 926902–14–9 and the POOL-predicted residues surrounding the conserved catalytic motif, Site 2. The hydrogen bonds are shown as pink arrows and salt bridges in a bluish-red line.

**926902-14-9** (Adenosine, 5′→P-ester with thiotetraphosphoric acid ([(HO) (HS)P(O)OP(O) (SH)]2O), P‴→5′-ester with uridine) (Figures 8A,B), the top hit, binds to the MTase POOL predicted NSP16 pocket surrounding the catalytic motif with an XP Gscore of -14.41 kcal/mol. It forms nine H-bonds and interactions with the residues G6829, L6898, D6897, N6899, D6912, C6913, Y6930, F6947, K6968, and N6996. It also forms two π- π interactions with Y6828 and F6947. The residues forming a pocket around the binding pose of **926902-14-9** are Y6828, G6829, D6830, M6840, N6841, K6844, G6869, G6871, S6872 D6897, L6898, N6899, D6912, C6913, A6914, D6928, M6929, Y6930, D6931, P6932, K6935, F6947, N6996, S6998, S6999, S7000, and E7001.

### Nucleocapsid protein (N-Cap)

#### POOL predicts multiple sites for the nucleocapsid protein

The SARS-CoV-2 N protein is divided into five domains: a predicted intrinsically disordered N-terminal domain (N-NTD); an RNA-binding domain; a predicted disordered central linker (LKR) within a Ser/Arg rich (S/R) domain; a dimerization domain; and a predicted disordered C-terminal domain (N-CTD). SARS-CoV has been shown to bind viral RNA *via* the N-NTD, N-CTD, and C-tail domains (Huang et al., 2004; Chen et al., 2007; Takeda et al., 2008). It has been reported that the LKR’s SR-rich region regulates N protein oligomerization upon phosphorylation (Peng et al., 2008) and the N-protein self-association is required for viral RNP assembly (Luo et al., 2006). The N-CTD has been shown to play a direct role in N protein dimerization and oligomerization (Luo et al., 2006; Yu et al., 2006; Chen et al., 2007; Takeda et al., 2008). Some important residues that interact with antiviral compounds from the RNA Binding domain are F66, R68, G69, Y123, I131, W132, V133, and A134 (Tatar et al., 2021).

**FIGURE 9:**
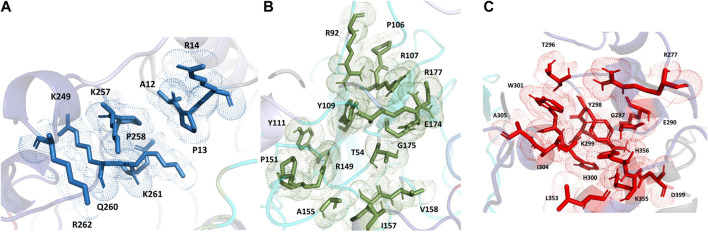
The different POOL-predicted sites for the SARS-CoV-2 Nucleocapsid protein. POOL-predicted sites for the full-length SARS-CoV-2 Nucleocapsid protein built on I-TASSER. **(A)** Site 1 shown in blue, **(B)** Site 2 in green and **(C)** Site 3 in red.

**FIGURE 10:**
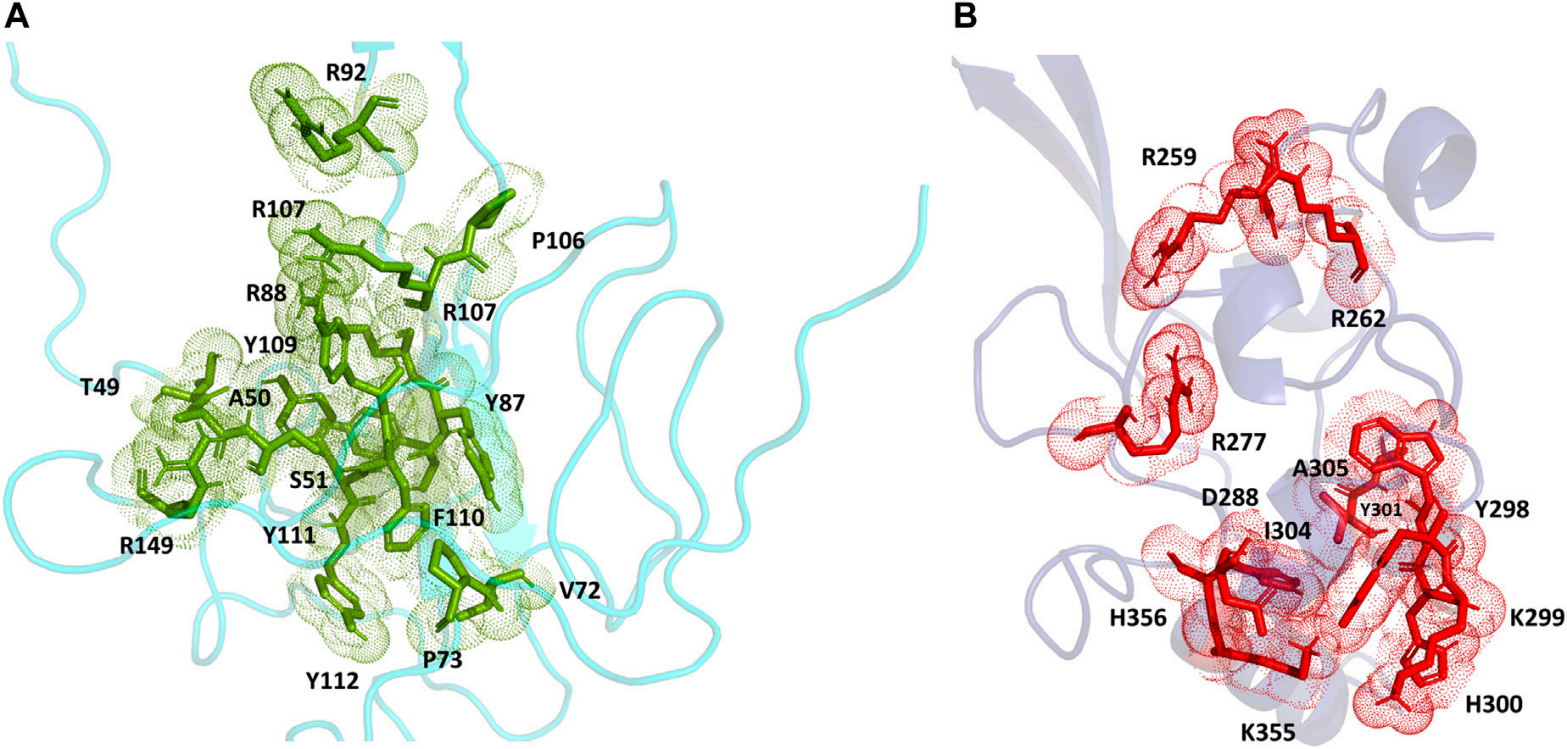
**(A)** The POOL-predicted residues for the SARS-CoV-2 Nucleocapsid protein N-terminal model built in YASARA **(B)** The POOL-predicted residues for the SARS-CoV-2 Nucleocapsid protein C-terminal structure from the PDB (PDB ID: 7DE1).

For the full-length nucleocapsid protein model POOL predicted three distinct clusters of residues in surface pockets. Site 1 (Figure 9A) consists of residues A12, P13, R14, K249, K257, Q260, K261, and R262, Site 2 (Figure 9B) consists of T54, R92, R107, Y109, Y111, R149, P151, A155, I157, V158, E174, G175, R177, G178, G179 and A311, while Site 3 (Figure 9C) consists of R259, R277, G287, E290, T296, Y298, K299, H300, W301, I304, A305, L353, K355, H356, and D399. For the N-terminus model of Nucleocapsid protein, POOL predicts the following residues (Figure 10): T49, A50, S51, V72, P73, Y86, Y87, R88, R92, R107, Y109, F110, Y111, Y112, and R149. For the C-terminus Nucleocapsid protein structure (PDB ID: 7DE1), the POOL predicted residues are (Figure 10): R259, R262, R277, D288, Y298, K299, H300, W301, I304, A305, K355, and H356.

For the SARS-CoV-2 N-terminal domain of the nucleocapsid protein, it has been reported that residues R92, R107, Y109 and R149 interact with the RNA (Dinesh et al., 2020); these residues are included in the POOL predictions for both the full-length N protein (Site 2) and the N-terminus model structure. We note that the POOL predictions for Site three for the full-length nucleocapsid model and for the C-terminal Nucleocapsid structure (PDB: 7DE1) are in good agreement.

#### Potential SARS-CoV-2 full length nucleocapsid protein inhibitors that bind to the NCap POOL-predicted site 1

Based on our docking studies and analysis, the potential ligand candidates that bind to full length Nucleocapsid protein POOL-predicted site one are ligands from the Enamine Covid library, Life Chemicals SARS-CoV-2 library and ZINC Database with ID# ZINC000085537017 **(**Cangrelor); Z1455181379 ((3-(1-(2,4-difluorophenyl)-2,5-dioxoimidazolidin-4-yl)propanoyl)proline); Z57170530 (4-hydroxy-3-((5-hydroxy-7-oxo-7,8-dihydro-1l3-chromen-6-yl))-2H-chromen-2-one); F0916-5053 (N (3chlorobenzyl)-4 [4-oxo-2 [(2-oxo-2{[3 (trifluoromethyl)phenyl]amino}ethyl)thio]quinazolin-3(4H)yl]butanamide); and Z1444935835 (3-{[2-(6-fluoro-1H-indol-3-yl)acetamido]methyl}benzoic acid), (Supplementary Table S4). Supplementary Table S4 shows the Glide docking method, docking scores, MM-GBSA scores along with the details about ligand-protein interactions. The list consists of mainly ligands from the Enamine Covid library, Life Chemicals SARS-CoV-2 library and ZINC Database. The docking score ranges for Extra Precision (XP) docking on Glide were -15 to -9 kcal/mol. The interactions between the ligand-protein complex are shown in the Supplementary Figure S6 and listed in Supplementary Table S4. All the compounds bind to the Ncap POOL-predicted sites and important H-bond interactions are observed with the residues R177, Q260, K261 and W301. Important π- π interactions are observed with W301.

ZINC000085537017 **(**Cangrelor), the top hit, is an ATP mimic and ubiquitous binder to multiple sites and targets. The second-best scoring hit, Z1455181379 (3-(1-(2,4-difluorophenyl)-2,5-dioxoimidazolidin-4-yl)propanoyl)proline) (Supplementary Figure S9), binds to full length Nucleocapsid protein at the POOL-predicted site 1 with an XP GScore of -11.05. It forms three hydrogen bonds with R177, T263, and A264. The residues forming a pocket around the binding pose of Z1455181379 are G175, R177, Q260, K261, R262, T263, A264, V270, F274, R277, F286, G287, L291, G295, T296, and W301.

#### Potential SARS-CoV-2 full length nucleocapsid protein inhibitors that bind to the NCap POOL-predicted site 2

Based on our docking studies and analysis, the potential ligand candidates that bind to full length Nucleocapsid protein POOL predicted site two are ligands from the ZINC Database with ID# ZINC000028467879 **(**Ceftriaxone), ZINC000004468778 (Cefixime), ZINC000003989268 (Ceftaroline Fosamil), ZINC000001540998 (Pemetrexed), and ZINC000004468778_2 (Cefixime), (Supplementary Table S5). Supplementary Table S5 shows the Glide docking method, docking scores, and MM-GBSA scores, along with the details about ligand-protein interactions. The docking score ranges for Extra Precision (XP) docking on Glide were between −15 and −9 kcal/mol. The interactions between the ligand-protein complex are shown in Supplementary Figure S7 and listed in Supplementary Table S5. All the compounds bind to the Ncap POOL predicted site two and important H-bond interactions are observed with the residues G175, R177, Q260, K261 and W301. Important π- π and π-cation interactions were observed with W301, R177, and K261.

ZINC000028467879 **(**Ceftriaxone), (Supplementary Figure S9) the top hit, binds to full length Nucleocapsid protein at the POOL predicted site 2 with an XP GScore of −11.53 kcal/mol. It forms five H-bond interactions with the residues S176, R177, Q260, T263 and A264. The residues forming a pocket around the binding pose of ZINC000028467879 are T54, A155, A156, V158, G175, S176, R177, Q260, K261, R262, T263, A264, V270, F274, R277, L291, G295, T296, TW301, A305, A308, P309, S310, and A311.

#### Potential SARS-CoV-2 full length nucleocapsid protein inhibitors that bind to the NCap POOL predicted site 3

Based on our docking studies and analysis, the potential ligand candidates that bind to full length Nucleocapsid protein POOL-predicted site three are ligands from the DrugBank and ZINC Database with ID# DB02738 **(**Adenosine-5′-Pentaphosphate), ZINC000085537017 (Cangrelor), DB03732 (Etheno-Nadp), DB04158 (6-(adenosine tetraphosphate-methyl)-7,8-dihydropterin), and DB02355 (Adenosine-5′-Rp-Alpha-Thio-Triphosphate), (Supplementary Table S6). Supplementary Table S6 shows the Glide docking method, docking scores, MM-GBSA scores, along with the details about ligand-protein interactions. The docking score ranges for Extra Precision (XP) docking on Glide were between -19 and -14 kcal/mol. The interactions between the ligand-protein complex are shown in Supplementary Figure S8 and listed in Supplementary Table S6. All the compounds bind to the Ncap POOL predicted site three and important H-bond interactions are observed with the residues G175, R177, Q260, K261 and W301. Important π- π and π-cation interactions were observed with W301, and R177. These compounds, as might be anticipated, are nucleotide-like.

## Conclusion

Some new insights into the functioning of three viral proteins have emerged. The nucleophilic C145 of the main protease is assisted by strong electrostatic coupling to H41, H163, and H164, so that it can be deprotonated and available to affect nucleophilic attack at neutral pH. H41, which exchanges a proton with the thioester intermediate, is assisted by strong coupling to C145 and H164. K6968, the catalytic base of the RNA methyltransferase, has a significant population of its deprotonated state at neutral pH, and therefore is able to act as a Brønsted base, through strong coupling to K6844 and Y6845. Its strength of basicity is enhanced by strong coupling to two acidic residues, D6928 and E7001. These two catalytic acidic residues of the RNA methyltransferase are strongly coupled to each other; the buffer range of D6928 is also expanded through strong coupling to H6867.

Multiple sites of likely biochemical significance are predicted for each of the three proteins, with multiple examples of ligands that may bind and interact with these sites. These sites represent alternative targets for the design of ligands to serve as chemical probes or inhibitors for these viral proteins.

## Data Availability

The original contributions presented in the study are included in the article/Supplementary Material, further inquiries can be directed to the corresponding author.
