# Supplementary material for: Identification and characterization of alternative sites and molecular probes for SARS-CoV-2 target proteins
Source: Front Chem. 2022 Oct 31;10:1017394. doi: 10.3389/fchem.2022.1017394 (PMC9659918; doi:10.3389/fchem.2022.1017394)
Supplement: Supplementary file 1 [file DataSheet1.docx]

Identification of alternative sites and molecular probes for SARS-CoV-2 target proteins

Suhasini M. Iyengar^1^, Kelly K. Barnsley^1^, Hoang Yen Vu^1^, Ian Jef A. Bongalonta^1^, Alyssa S. Herrod^1^, Jasmine A. Scott^1^, Mary Jo Ondrechen^1^

^1^Department of Chemistry and Chemical Biology, Northeastern University, Boston, MA-02115, USA

Supplementary Material

List of figures

| S. No. | Figure | Page No. |
| --- | --- | --- |
| **S1** | Protein reliability reports generated on Maestro for the SARS-CoV-2 target structures. | 3 |
| **S2** | Images from QMean Structure validation server showing the local quality of the sequence for the Nucleocapsid protein homology models | 4 |
| **S3** | Hits bound to the monomer of the SARS-CoV-2 MPro at the POOL predicted secondary site. | 5 |
| **S4** | CAS hits bound to the dimer of the SARS-CoV-2 MTase at the POOL predicted conserved catalytic motif. | 6 |
| **S5** | CAS hits bound to the dimer of the SARS-CoV-2 MTase at the POOL predicted nsp16 pocket surrounding conserved catalytic motif. | 7 |
| **S6** | Hits bound to the SARS-CoV-2 full-length Nucleocapsid protein at the POOL predicted Site 1. | 8 |
| **S7** | Hits bound to the SARS-CoV-2 full-length Nucleocapsid protein at the POOL predicted Site 2. | 9 |
| **S8** | Hits bound to the SARS-CoV-2 full-length Nucleocapsid protein at the POOL predicted Site 3. | 10 |
| **S9** | Top Hits bound to the SARS-CoV-2 full-length Nucleocapsid protein at the POOL predicted Site 1 and 2. | 11 |
|  | List of Tables |  |
| ST. No. | Table | Page No. |
| S1 | Predicted Top binders for the SARS-CoV-2 MPro POOL predicted secondary site | 12 |
| S2 | Predicted Top binders for the SARS-CoV-2 MTase POOL predicted site containing the conserved catalytic K-D-K-E motif | 14 |
| S3 | Predicted Top binders for the SARS-CoV-2 MTase POOL predicted NSP16 pocket surrounding the catalytic motif | 16 |
| S4 | Predicted Top binders for the SARS-CoV-2 full-length Nucleocapsid Protein POOL predicted Site 1 | 20 |
| S5 | Predicted Top binders for the SARS-CoV-2 full-length Nucleocapsid Protein POOL predicted Site 2 | 22 |
| S6 | Predicted Top binders for the SARS-CoV-2 full-length Nucleocapsid Protein POOL predicted Site 3 | 25 |


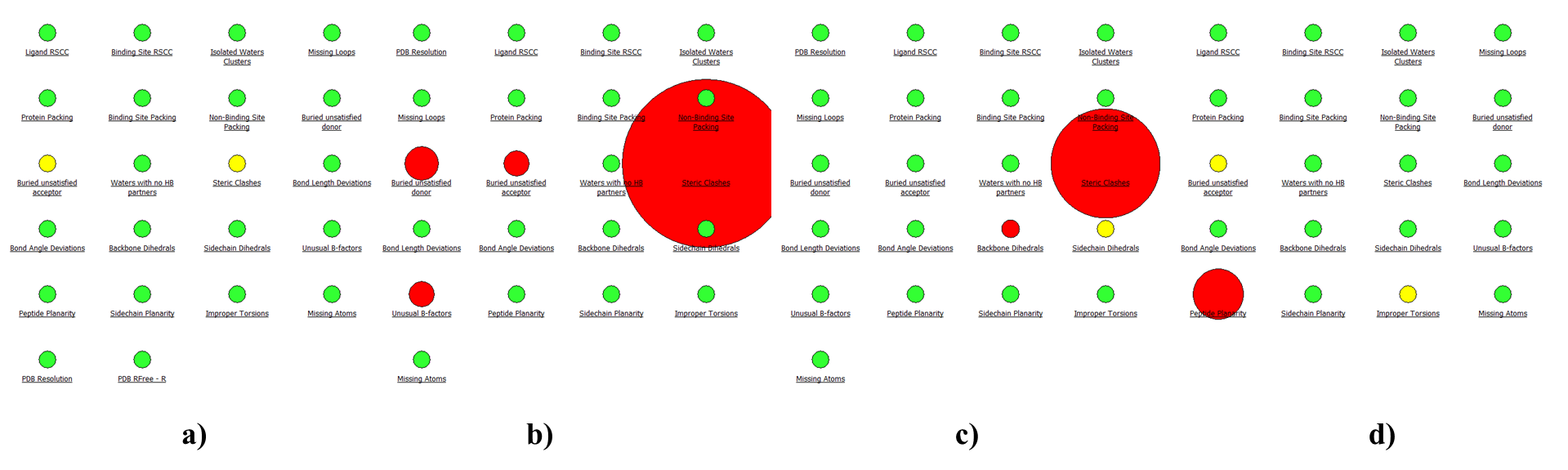


**Supplementary Figure** **S1**: Protein reliability reports generated on Maestro for the SARS-CoV-2 target structures. The circles are a visual representation for the metrics of protein reliability with the radius of the circle indicating the magnitude of the metric. Circles colored red indicate a deviation from acceptable values beyond a certain cutoff. Circles colored yellow indicates when a cutoff is exceeded by one. Green circles represent a small or minimal deviation. a) Main Protease (PDB ID: 6LU7) b) Methyltransferase (PDB ID: 6W4H) c) Nucleocapsid protein C-terminus (PDB ID: 7DE1) d) Nucleocapsid protein full-length model.


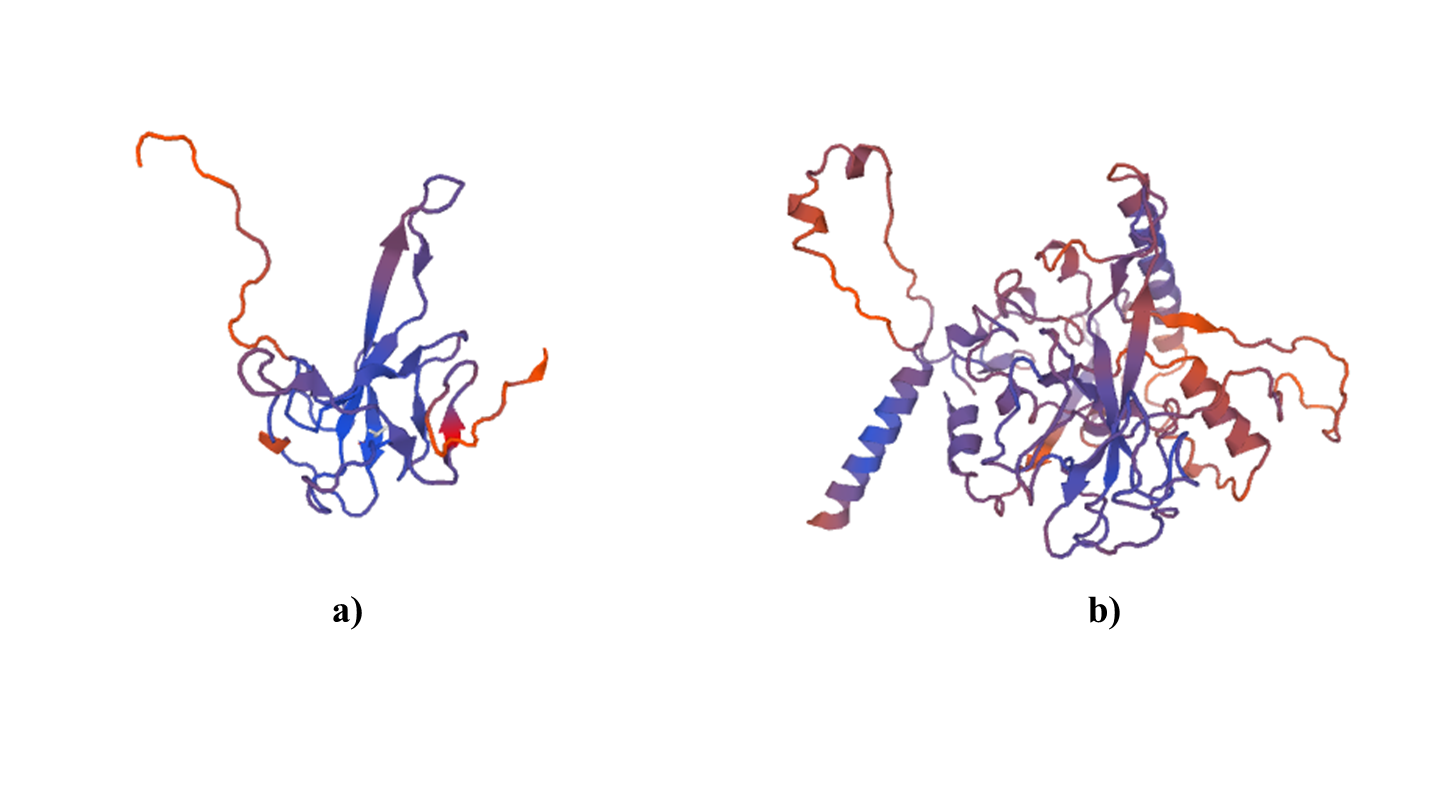


**Supplementary Figure** **S2:** Images from QMean Structure validation server showing the local quality of the sequence for the Nucleocapsid protein homology models. Blue indicates better quality regions; orange indicates lower quality regions. a) N Terminal model b) Full-length model

**
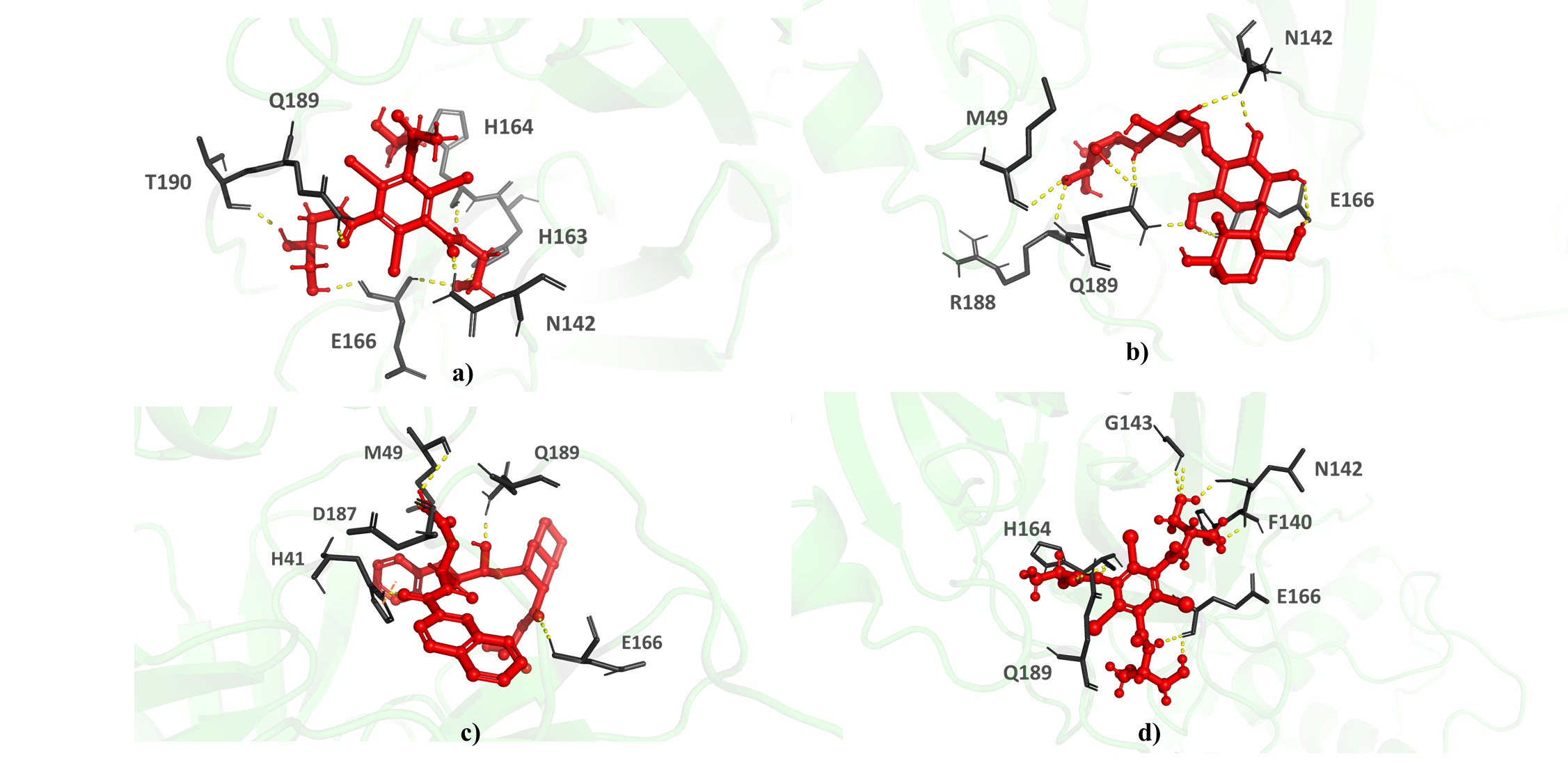
**

**Supplementary Figure** **S3:** Hits bound to the monomer of the SARS-CoV-2 M^Pro^ at the POOL predicted recognition (or secondary) site. The protein is shown in green cartoon representation, ligand in red with the residues in gray. The hydrogen bonds are shown as yellow dashes and π- π stacking interactions as orange dashes a) Ioxilan b) Acarbose c) Saquinavir d) Iopamidol


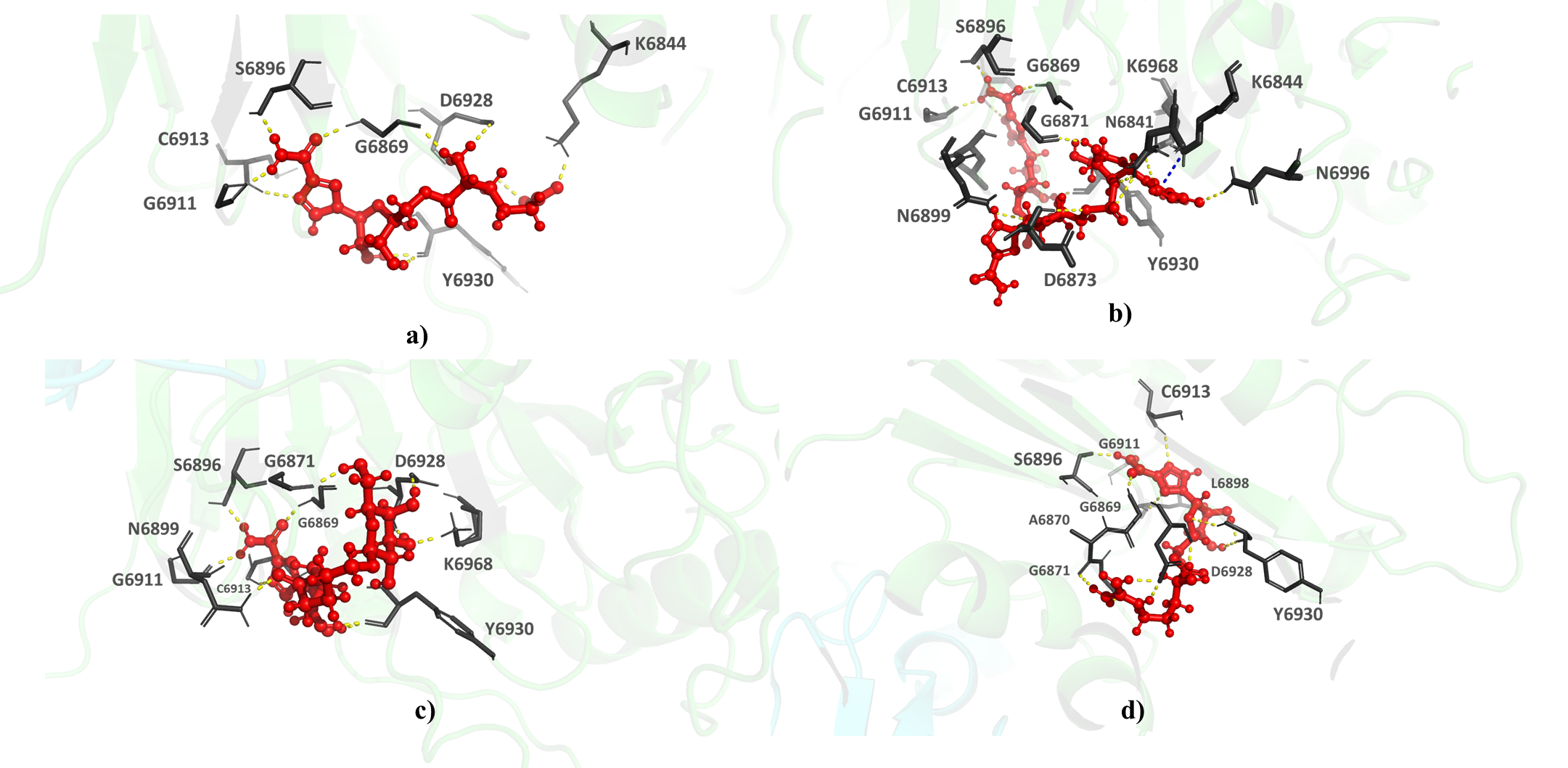


**Supplementary Figure** **S4:** CAS hits bound to the SARS-CoV-2 M^Tase^ at the POOL predicted site containing the conserved catalytic motif. The protein is shown in cartoon representation in green, ligand in red with the residues in gray. The hydrogen bonds are shown as yellow dashes. a) 435297-58-8 b) 1312805-81-4 c) 1002334-92-0 d) 435297-32-8.


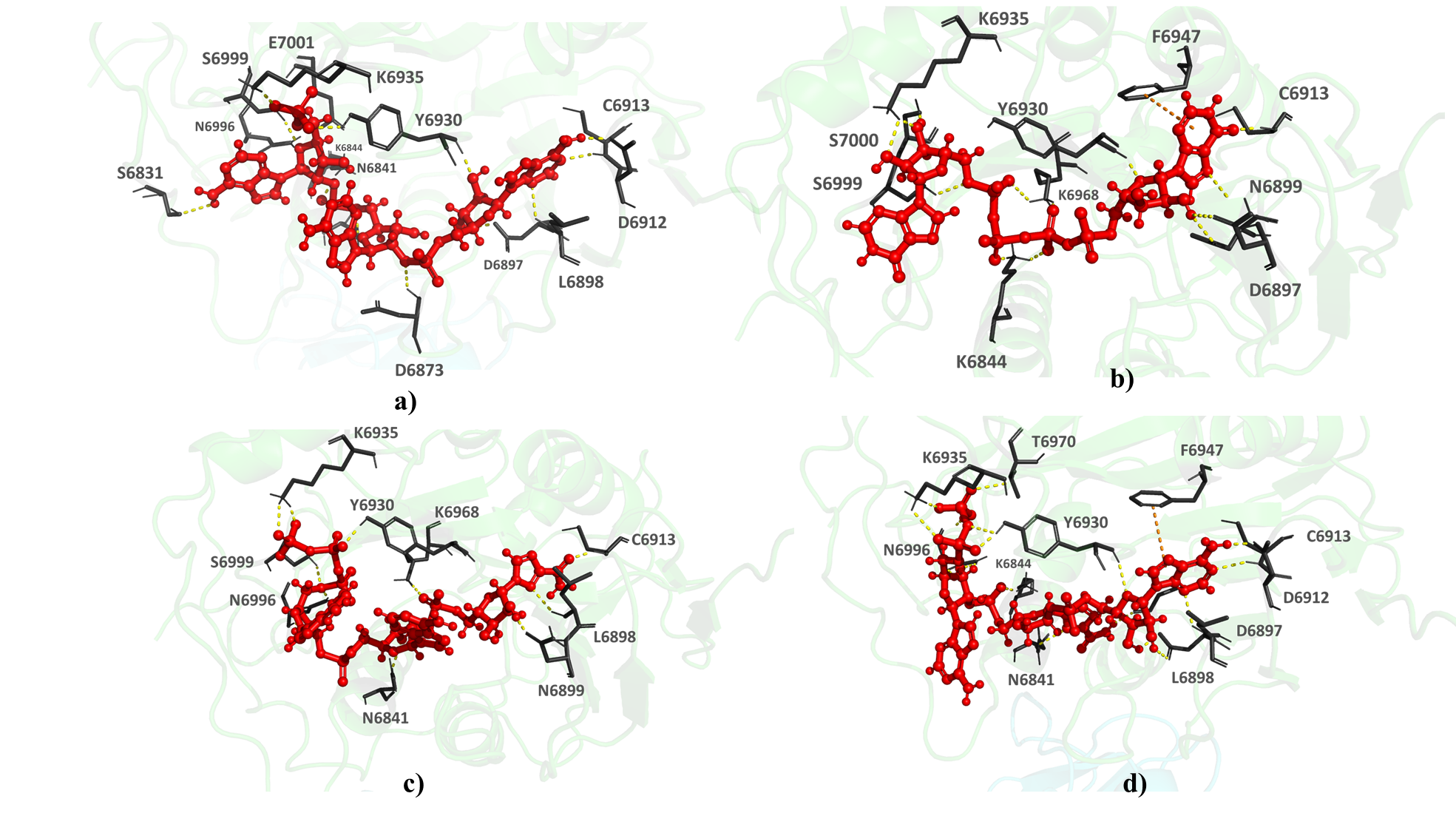


**Supplementary Figure** **S5:** CAS hits bound to the SARS-CoV-2 M^Tase^ at the POOL predicted nsp16 pocket surrounding conserved catalytic motif. The protein is shown in cartoon representation in green, ligand in red with the residues in gray. The hydrogen bonds are shown as yellow dashes. a) 162754-90-7 b) 188560-02-3 c) 217807-08-4 d) 217807-10-8.


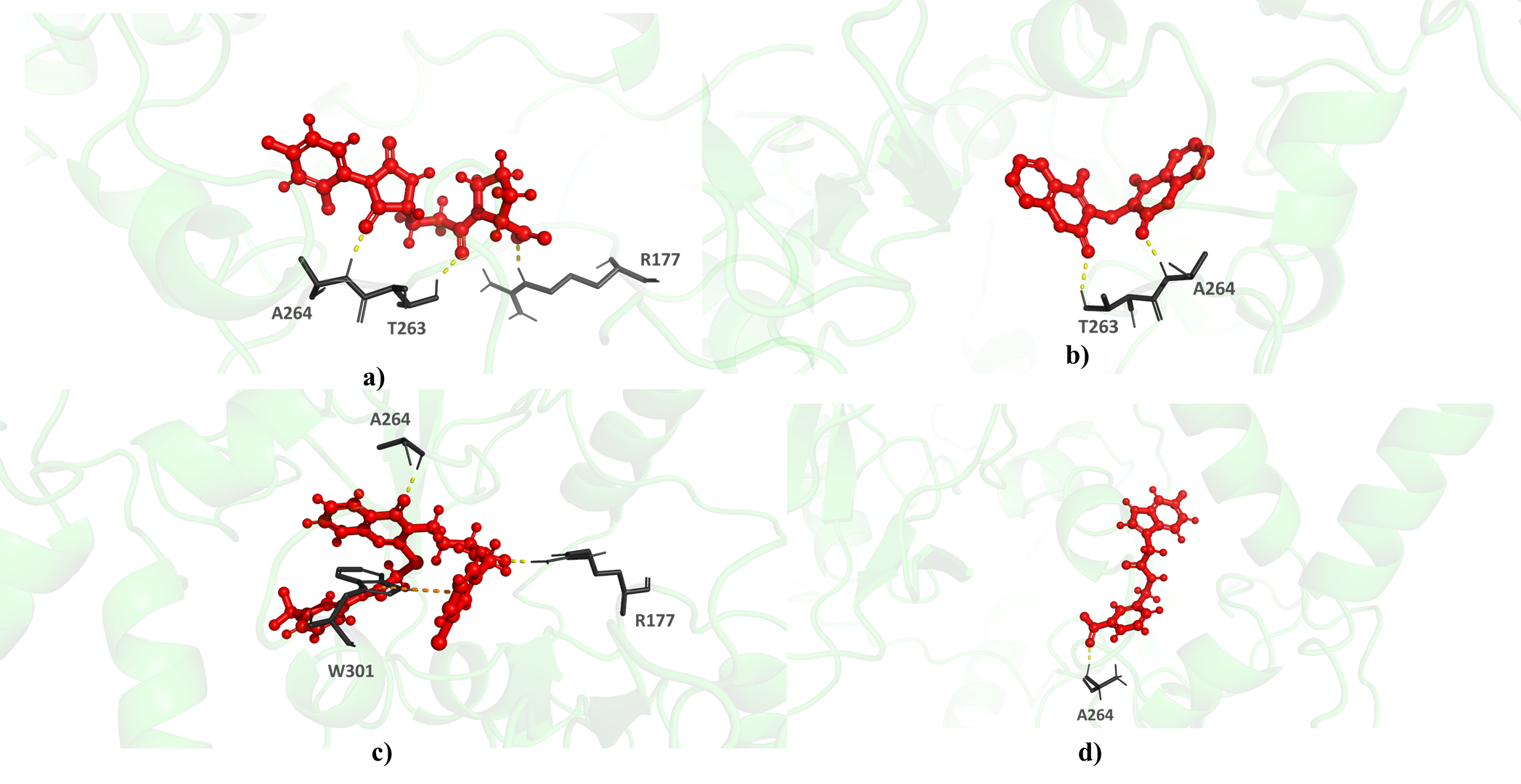


**Supplementary Figure** **S6**: Hits bound to the SARS-CoV-2 full-length Nucleocapsid protein at the POOL predicted Site 1. The protein is shown in cartoon representation with the Ncap protein green, ligand in red with the Ncap residues forming a H-bond in gray. The hydrogen bonds are shown as yellow dashes, π- π interactions are shown as orange dashes. a) Z1455181379 b) Z57170530

c) F0916-5053 d) Z1444935835


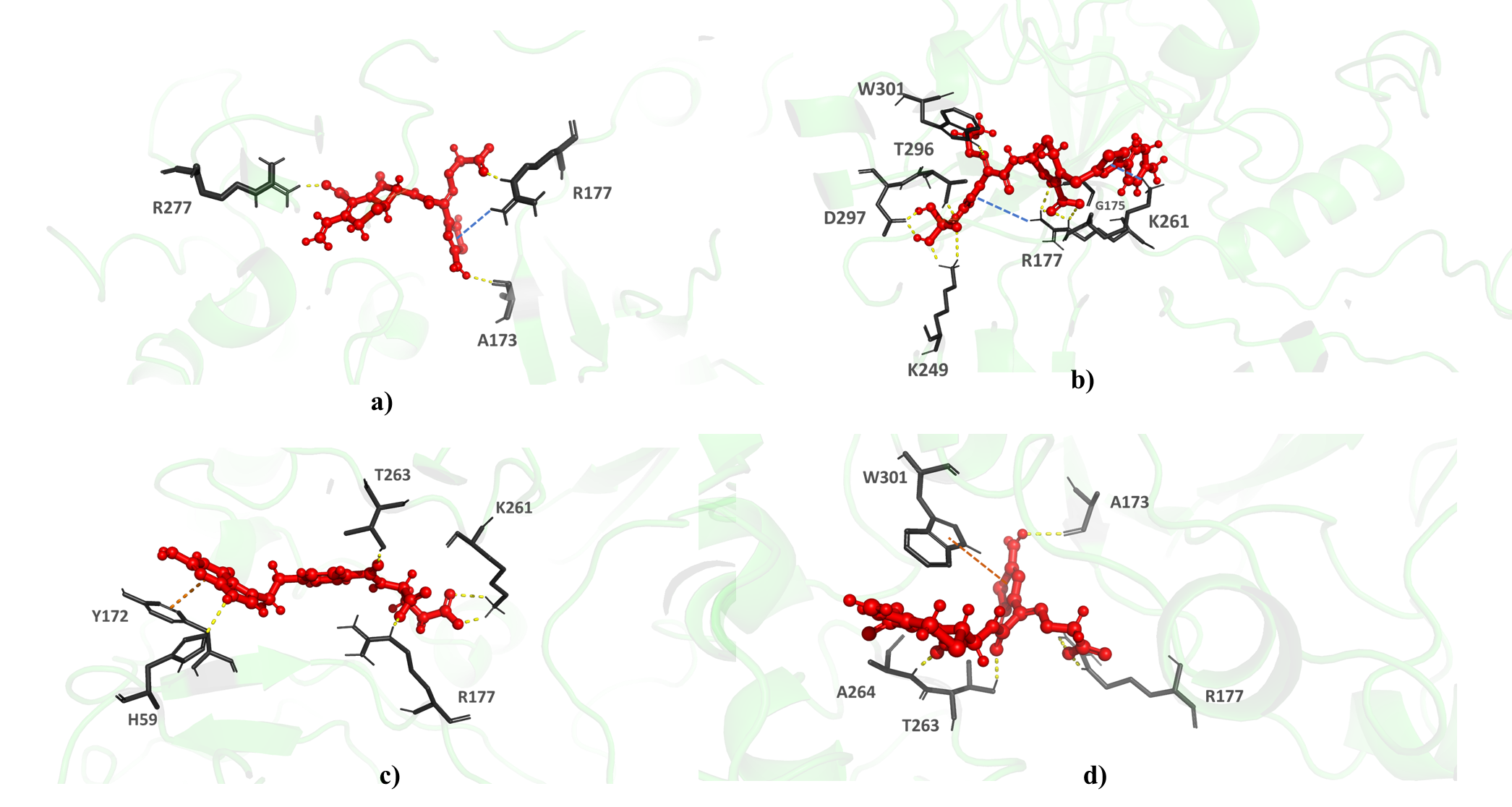


**Supplementary Figure** **S7**: Hits bound to the SARS-CoV-2 full-length Nucleocapsid protein at the POOL predicted Site 2. The protein is shown in cartoon representation with the Ncap protein green, ligand in red with the Ncap residues forming a H-bond in gray. The hydrogen bonds are shown as yellow dashes, π- π interactions are shown as orange dashes. a) ZINC000004468778 b) ZINC000003989268 c) ZINC000001540998 d) ZINC000004468778-2


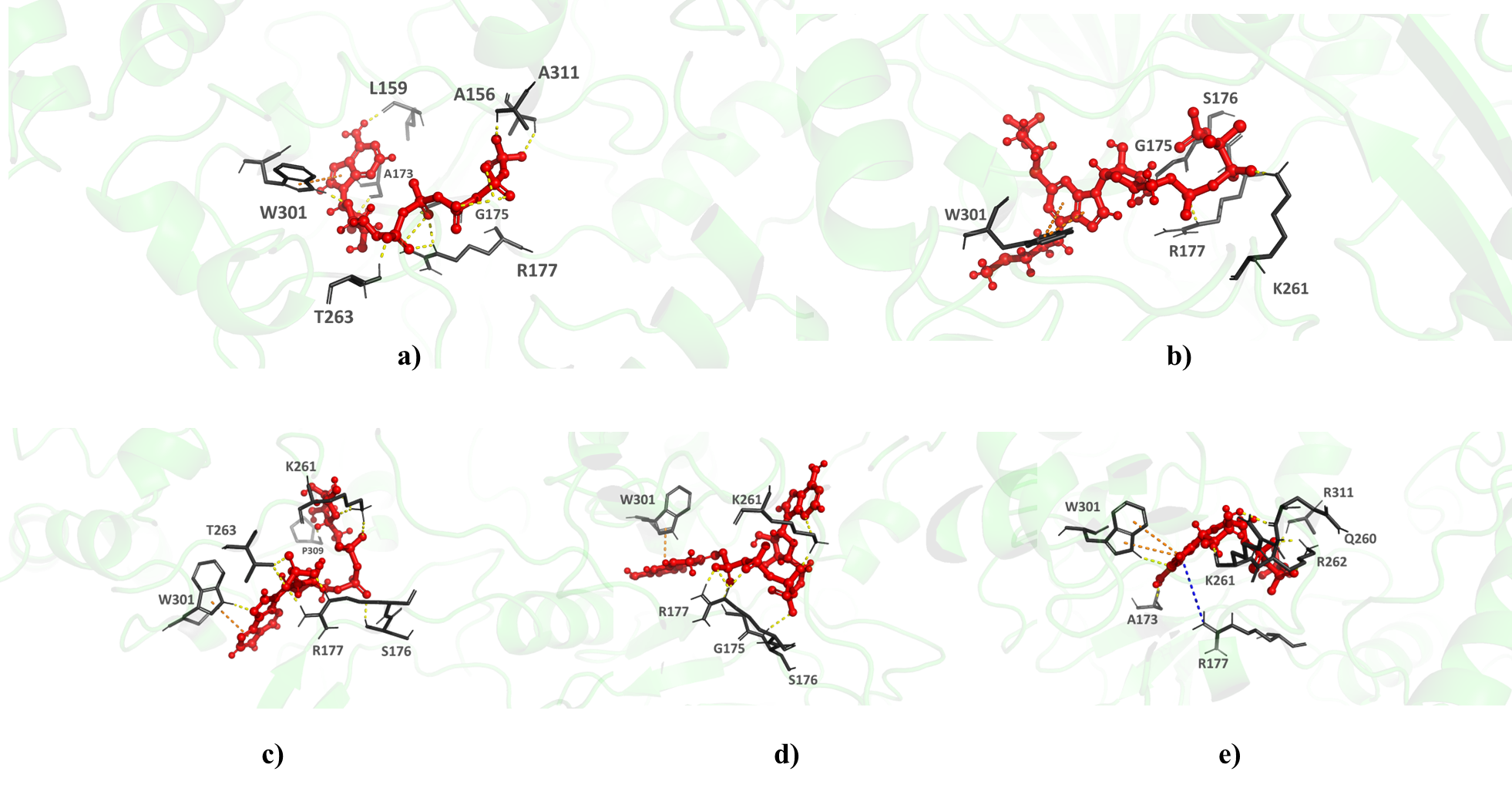


**Supplementary Figure** **S8**: Hits bound to the SARS-CoV-2 full-length Nucleocapsid protein at the POOL predicted Site 3. The protein is shown in cartoon representation with the Ncap protein green, ligand in red with the Ncap residues forming a H-bond in gray. The hydrogen bonds are shown as yellow dashes, π- π interactions are shown as orange dashes. a) DB02738 b) ZINC000085537017 c) DB03732 d) DB04158 e) DB02355


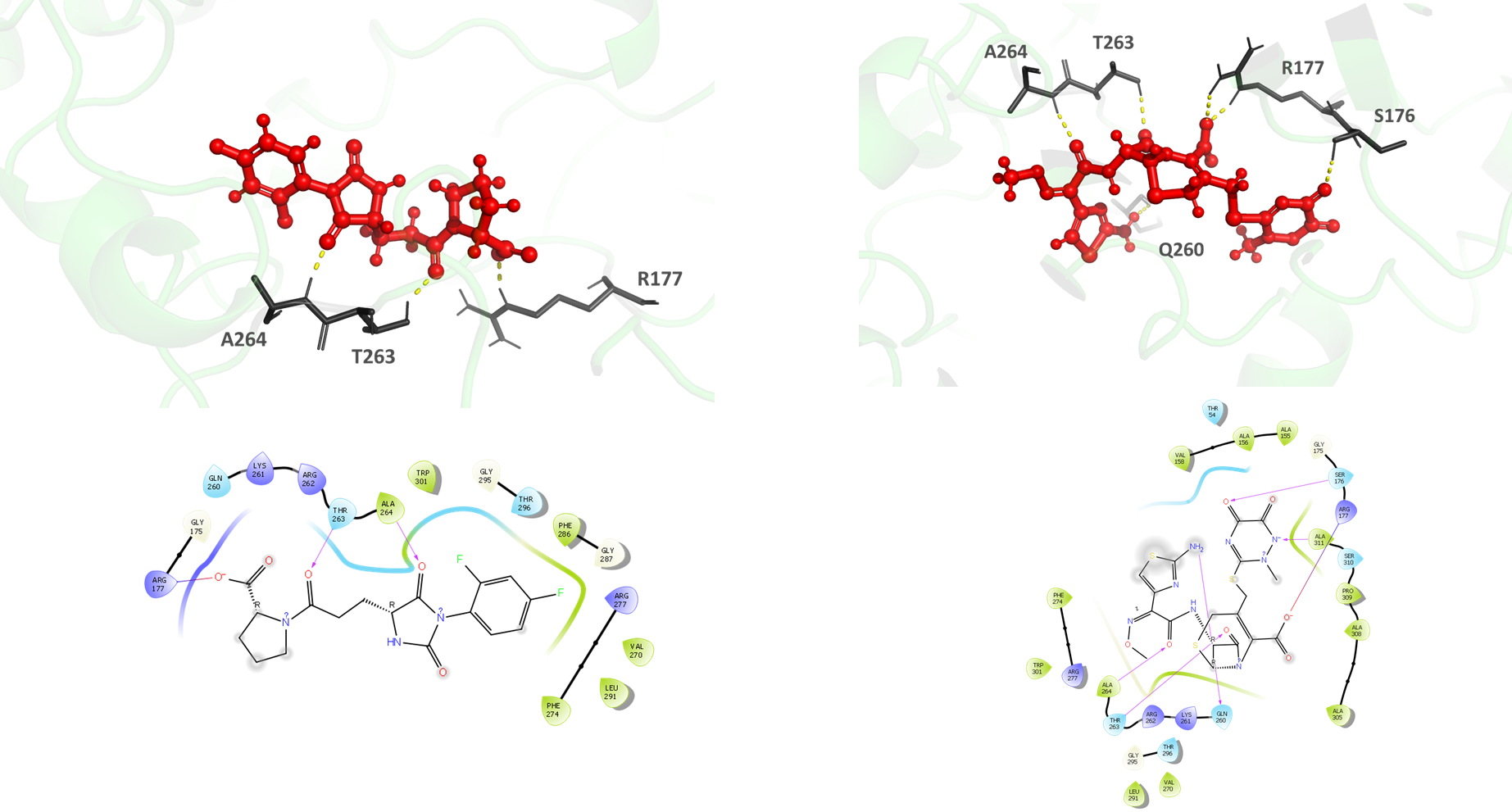


**Supplementary Figure** **S9: (A, left):** Z1455181379 bound to the SARS-CoV-2 full-length Nucleocapsid protein at the POOL-predicted Site 1. (**B, right):** ZINC000028467879 bound to the SARS-CoV-2 full-length Nucleocapsid protein at the POOL-predicted Site 2. Top: The protein backbone is shown in cartoon representation with the Ncap protein green, ligand in red with the Ncap residues forming a H-bond in gray. The hydrogen bonds are shown as yellow dashes, π- π interactions are shown as orange dashes. Bottom: Ligand interaction diagram where the hydrogen bonds are shown as pink arrows, salt bridges in a bluish-red line, and π- cation interactions as red lines.

**Table S1 : Predicted Top binders for the SARS-CoV-2 M^Pro^ POOL predicted secondary site**

| **Ligand** | **ID Number** | **IFD Docking Score (kcal/mol)** | **Interactions** | **Residues forming the pocket** |
| --- | --- | --- | --- | --- |
| Epigallocatechin Gallate | **989-51-5** | -14.12 | **H-Bond:** Thr26, His41, Tyr54, Asn142, His163, His164, Glu166, Gln189  **π- π:** His41 | Thr25, Thr26, Leu27, His41, Cys44, Met49, Pro52, Tyr54, Phe140, Leu141, Asn142, Gly143, Ser144, Cys145, His163, His164, Met165, Glu166, Asp187, Arg188,Gln189. |
| Ioxilan | **107793-72-6** | -13.32 | **H-Bond:** Asn142, His163, His164, Glu166, Gln189, Thr190  **Halogen Bond:** Glu166----I | His41, Met49, Tyr54, Phe140, Leu141, Asn142, Gly143, Ser144, Cys145, His163, His164, Met165, Glu166, Leu167, Pro168, His172, Phe181, Asp187, Arg188,Gln189, Thr190, Ala191, Gln192. |
| Acarbose | **56180-94-0** | -12.61 | **H-Bond:** Asn142, Glu166, Gln189 | Pro39, His41, Met49, Tyr54, Leu141, Asn142, Gly143, Cys145, His163, His164, Met165, Glu166, Leu167, Pro168, Gly170, Phe181, Asp187, Arg188,Gln189, Ala191. |
| Saquinavir | **149845-06-7** | -12.35 | **H-Bond:** Asn142, Asp187, Gln189  **π- π:** His164 | Thr25, Thr26, Leu27, His41, Val42, Cys44, Met49, Pro52, Cys85, Phe140, Leu141, Asn142, Gly143, Ser144, Cys145, His163, His164, Met165, Glu166, Leu167, His172, Ala173, Gly174, Thr175, Phe181, Tyr182, Phe185, Asp187, Arg188, Gln189. |
| Iopamidol | **62883-00-5 60166-93-0 66108-95-0** | -11.98 | **H-Bond:**  Phe140Asn142, His163, His164, Glu166, Gln189 | His41, Met49, Tyr54, Phe140, Leu141, Asn142, Gly143, Ser144, Cys145, His163, His164, Met165, Glu166, Leu167, Pro168, His172, Asp187, Arg188, Gln189 Thr190, Ala191, Gln192. |

**Table S2 : Predicted Top binders for the SARS-CoV-2 M^Tase^ POOL predicted site containing the conserved catalytic K-D-K-E motif**

| **Ligand** | **CAS- ID Number** | **XP Docking Score (kcal/mol)** | **MM-GBSA Scores(kcal/mol)** | | **Interactions** | **Residues forming the pocket** |
| --- | --- | --- | --- | --- | --- | --- |
| 1H-1,2,4-Triazole-3-carboxamide, 1-β-D-ribofuranosyl-, 5′-[6-hydrogen (2R)-2-aminohexanedioate | **435297-57-7** | -14.88 | -49.67 | | **H-Bond:**  Gly6869, Ser6896, Asp6897,Gly6911,  Cys6913, Tyr6930, Lys6968 | Asn6841, Lys6844, Phe6868, Gly6869, Ala6970, Gly6871, Ser6872, Ser6896, Asp6897, Leu6898, Asn6899, Gly6911, Asp6912, Cys6913, Val6916, Asp6928, Met6929, Tyr6930, Asp6931, Phe6947, Lys6968 |
| 1H-1,2,4-Triazole-3-carboxamide, 1-β-D-ribofuranosyl-, 5′-[6-hydrogen (2S)-2-aminohexanedioate | **435297-58-8** | -14.75 | | -53.28 | Lys6844, Gly6869, Ser6896, Gly6911, Cys6913, Asp6928, Tyr6930 | Asn6841, Lys6844, Phe6868, Gly6869, Gly6871, Ser6872, Ser6896, Asp6897, Leu6898, Asn6899, Gly6911, Asp6912, Cys6913, Val6916, Asp6928, Met6929, Tyr6930, Asp6931, Phe6947, Glu7001 |
| Adenosine, 1′-[3-(aminocarbonyl)-1H-1,2,4-triazol-1-yl]-1′-de(6-amino-9H-purin-9-yl)adenylyl-(2′→5′)-1′-[3-(aminocarbonyl)-1H-1,2,4-triazol-1-yl]-1′-de(6-amino-9H-purin-9-yl)adenylyl-(2′→5′)-1′-[3-(aminocarbonyl)-1H-1,2,4-triazol-1-yl]-1′-de(6-amino-9H-purin-9-yl)-(ACl)) | **1312805-81-4** | -14.51 | | -41.04 | Asn6841, Lys6844, Gly6869, Gly6871, Asp6873, Ser6896, Asn6899, Gly6911, Cys6913, Tyr6930, Lys6968, Asn6996 | Asn6841, Lys6844, Phe6868, Gly6869, Gly6871, Ser6872, Asp6873, Ser6896, Asp6897, Leu6898, Asn6899, Gly6911, Asp6912, Cys6913, Val6916, Asp6928, Met6929, Tyr6930, Asp6931, Pro6932, Phe6947, Thr6970, Lys6968, Asn6996, Ser6999, Glu7001 |
| 1H-1,2,4-Triazole-3-carboxamide, 1-[5-O-[5-(β-D-galactopyranosyloxy)-1-oxopentyl]-β-D-ribofuranosyl]-(ACl) | **1002334-92-0** | -14.41 | | -63.17 | Gly6869, Gly6871, Ser6896, Asn6899, Gly6911, Cys6913, Asp6928, Tyr6930, Lys6968 | Asn6841, Lys6844, Phe6868, Gly6869, Ala6870, Gly6871, Ser6872, Asp6873, Pro6978, Ser6896, Asp6897, Leu6898, Asn6899, Gly6911, Asp6912, Cys6913, Val6916, Asp6928, Met6929, Tyr6930, Asp6931, Phe6947, Lys6968 |
| L-Arginine, 5′-ester with 1-β-D-ribofuranosyl-1H-1,2,4-triazole-3-carboxamide- (9Cl) | **435297-32-8** | -13.47 | | -72.94 | Gly6869, Ala6870, Gly6871, Ser6896, Leu6898, Gly6911, Cys6913, Asp6928, Tyr6930 | Asn6841, Lys6844, Tyr6845, His6867, Phe6868, Gly6869, Ala6870, Gly6871, Ser6872, Ala6877, Pro6878, Gly6879, Thr6880, Ser6896, Asp6897, Leu6898, Asn6899, Gly6911, Asp6912, Cys6913, Val6916, Asp6928, Met6929, Tyr6930, Asp6931, Phe6947, Lys6968 |

**Table S3: Predicted Top binders for the SARS-CoV-2 M^Tase^ POOL predicted NSP16 pocket surrounding the catalytic motif**

| **Ligand** | **CAS- ID Number** | **XP Docking Score (kcal/mol)** | **MM-GBSA Scores(kcal/mol)** | **Interactions** | **Residues forming the pocket** |
| --- | --- | --- | --- | --- | --- |
| Adenosine, 5′→P-ester with thiotetraphosphoric acid ([(HO)(HS)P(O)OP(O)(SH)]2O), P′′′→5′-ester with uridine | **926902-14-9** | -14.41 | -40.97 | **H-Bond:**  Gly6829, Asp6897, Leu6898, Asn6899, Asp6912, Cys6913, Tyr6930, Phe6947, Lys6968, Asn6996  **π- π:**  Tyr6828, Phe6947 | Tyr6828, Gly6829, Asp6830, Met6840, Asn6841, Lys6844, Gly6869, Gly6871, Ser6872 Asp6897, Leu6898, Asn6899, Asp6912, Cys6913, Ala6914, Asp6928, Met6929, Tyr6930, Asp6931, Pro6932, Lys6935, Phe6947, Asn6996, Ser6998, Ser6999, Ser7000, Glu7001 |
| β-D-arabino-Adenosine, 5′-O-phosphonoadenylyl-(2′→5′)-adenylyl-(2′→5′)- (9Cl) | **162754-90-7** | -14.08 | -41.52 | **H-Bond:**  Ser6831, Asn6841, Lys6844, Asp6897, Leu6898, Asp6912, Cys6913, Tyr6930, Lys6935, Asn6996, Ser6999, Glu7001 | Tyr6828, Asp6830, Ser6831, Met6840, Asn6841, Lys6844, Phe6868, Gly6869, Gly6871, Ser6872, Asp6873, Asp6897, Leu6898, Asn6899, Gly6911, Asp6912, Cys6913, Ala6914, Asp6928, Met6929, Tyr6930, Asp6931, Pro6932, Lys6935, Lys6968, Thr6970, Asn6996, Ser6998, Ser6999, Ser7000, Glu7001 |
| Inosine 5′-(pentahydrogen tetraphosphate), P′′′→5′-ester with inosine | **188560-02-3** | -13.54 | -11.45 | **H-Bond:**  Lys6844, Asp6897, Asn6899, Cys6913, Tyr6930, Lys6935, Phe6947, Lys6968, Asn6996, Ser6999, Ser7000  **π- π:**  Phe6947 | Tyr6828, Gly6829, Asp6830, Ser6831, Met6840, Asn6841, Lys6844, Gly6869, Ser6872, Asp6873, Asp6897, Leu6898, Asn6899, Asp6912, Cys6913, Asp6928, Met6929, Tyr6930, Lys6935, Phe6947,  Lys6968, Thr6970, Glu6971, Asn6996, Ser6998, Ser6999, Ser7000, Glu7001 |
| Adenosine, 5′-O-[hydroxy [[hydroxy(phosphonooxy) phosphinyl]oxy] phosphinyl]adenylyl-(2′→5′)-adenylyl-(2′→5′)-1′-[3-(aminocarbonyl)-1H-1,2,4-triazol-1-yl]-1′-de(6-amino-9H-purin-9-yl)- (9Cl) | **217807-08-4** | -13.48 | -7.64 | **H-Bond:**  Asn6841, Leu6898, Lys6844, Asn6899, Cys6913, Tyr6930, Lys6935, Lys6968, Asn6996, Ser6999 | Tyr6828, Met6840, Asn6841, Lys6844, Phe6868, Gly6869, Gly6871, Ser6872, Asp6873, Ser6896, Asp6897, Leu6898, Asn6899, Gly6911, Asp6912, Cys6913, Asp6928, Met6929, Tyr6930, Pro6932, Lys6935, Lys6968, Thr6970, Asn6996, Ser6999, Ser7000, Glu7001 |
| Adenosine, 5′-O-[hydroxy(phosphonooxy)phosphinyl]adenylyl-(2′→5′)-1′-[3-(aminocarbonyl)-1H-1,2,4-triazol-1-yl]-1′-de(6-amino-9H-purin-9-yl)adenylyl-(2′→5′)- (9Cl) | **217807-10-8** | -13.43 | -25.49 | **H-Bond:**  Asn6841, Lys6844, Asp6897, Leu6898, Asp6912, Cys6913, Tyr6930, Lys6935, Phe6947, Lys6968, Thr6970, Asn6996  **π- π:**  Phe6947 | Tyr6828, Met6840, Asn6841, Lys6844, His6867, Gly6869, Ala6870, Gly6871, Ser6872, Asp6873, Pro6878, Gly6879. Asp6897, Leu6898, Asn6899, Gly6911, Asp6912, Cys6913, Asp6914, Asp6928, Met6929, Tyr6930, Asp6931, Pro6932, Lys6935, Phe6947, Lys6968, Thr6970, Glu6971, His6972, Asn6996, Ser6999, Ser7000, Glu7001 |

**Table S4: Predicted Top binders for the SARS-CoV-2 full-length Nucleocapsid Protein POOL predicted Site 1**

| **Ligand** | **ID Number** | **XP Docking Score (kcal/mol)** | **MM-GBSA Scores(kcal/mol)** | **Interactions** | **Residues forming the pocket** |
| --- | --- | --- | --- | --- | --- |
| Cangrelor | **ZINC000085537017** | -15.91 | -59.91 | **H-Bond:**  Arg177, Gln260, Lys261, Trp301 Ala311 | Thr54, His59, Ala155, Ala156,Val158, Tyr172, Ala173, Glu174, Gly175, Ser176, Arg177, Gln260, Lys261, Arg262, Thr263, Ala264, Phe274, Thr296, Trp301, Pro302, Ala305, Ala308, Pro309, Ser310, Ala311, Ser312 |
| 3-(1-(2,4-difluorophenyl)-2,5-dioxoimidazolidin-4-yl)propanoyl)proline | **Z1455181379** | -11.05 | -58.81 | **H-Bond:**  Arg177, Thr263, Ala264 | Gly175, Arg177, Gln260, Lys261, Arg262, Thr263, Ala264, Val270, Phe274, Arg277, Phe286, Gly287, Leu291, Gly295, Thr296, Trp301 |
| 4-hydroxy-3-((5-hydroxy-7-oxo-7,8-dihydro-1l3-chromen-6-yl)methyl)-2H-chromen-2-one | **Z57170530** | -10.18 | -38.39 | **H-Bond:**  Thr263, Ala264 | Val158, Ala173, Glu174, Gly175, Arg177, Arg262, Thr263, Ala264, Val270, Phe274, Arg277, Phe286, Gly287, Leu291, Gly295, Thr296, Trp301, Ala305, Ala308 |
| N(3chlorobenzyl)-4[4-oxo-2[(2-oxo-2{[3(trifluoromethyl)phenyl]amino}ethyl)thio]quinazolin-3(4H)yl]butanamide | **F0916-5053** | -10.08 | -71.53 | **H-Bond:**  Arg177, Ala264  **π- π:**  Trp301 | Val158, Tyr172, Ala173, Glu174, Gly175, Arg177, Arg262, Thr263, Ala264, Val270, Phe274, Arg277, Phe286, Leu291, Gly295, Thr296, Trp301, Pro302, Ile304, Ala305, Phe307, Ala308, Pro309, Leu353 |
| 3-{[2-(6-fluoro-1H-indol-3-yl)acetamido]methyl}benzoic acid | **Z1444935835** | -9.98 | -47.74 | **H-Bond:**  Ala264 | Arg262, Thr263, Ala264, Phe274, Arg277, Phe286, Leu291, Gly295, Thr296, Trp301, Ile304, Ala305, Phe307, Ala308, Pro309, Ile337, Ile357 |

**Table S5: Predicted Top binders for the SARS-CoV-2 full-length Nucleocapsid Protein POOL predicted Site 2**

| **Ligand** | **ID Number** | **Docking Score (kcal/mol)** | **MM-GBSA Scores(kcal/mol)** | **Interactions** | **Residues forming the pocket** |
| --- | --- | --- | --- | --- | --- |
| Ceftriaxone | **ZINC000028467879** | -11.53 | -67.29 | **H-Bond:**  Ser176, Arg177, Gln260, Thr263, Ala264 | Thr54, Ala155, Ala156,Val158, Gly175, Ser176, Arg177, Gln260, Lys261, Arg262, Thr263, Ala264, Val270, Phe274, Arg277, Leu291, Gly295, Thr296, Trp301, Ala305, Ala308, Pro309, Ser310, and Ala311 |
| Cefixime | **ZINC000004468778** | -11.32 | -55.42 | **H-Bond:**  Ala173, Arg177, Arg277  **π- cation:** Arg177 | Val158, Ala173, Glu174, Arg177, Gln260, Lys261, Arg262, Thr263, Ala264, Val270, Phe274, Arg277, Phe286, Gly287, Leu291, Gly295, Thr296, Trp301, Ala305 |
| Ceftaroline Fosamil | **ZINC000003989268** | -11.12 | -67.73 | **H-Bond:**  Gly175, Arg177, Lys249, Lys261, Thr296, Asp297, Trp301  **π- cation:** Arg177, Lys261 | Thr54, His59, Ala155, Ala156, Tyr172, Ala173, Glu174, Gly175, Ser176, Arg177, Gln260, Lys261, Thr263, Ala264, Thr265, Thr296, Asp297, Trp301, Pro302, Ala305, Ala308, Ser310, Ala311, Ser312 |
| Pemetrexed | **ZINC000001540998** | -9.88 | -42.43 | **H-Bond:**  His59, Tyr172, Arg177, Thr263, Lys261 | His59, Tyr172, Ala173, Glu174, Gly175, Arg177, Gln260, Lys261, Thr263, Thr296, Asp297, Trp301, Pro302, Ala305 |
| Cefixime | **ZINC000004468778_2** | -9.60 | -50.11 | **H-Bond:**  Ala173, Arg177, Thr263, Ala264  **π- π:**  Trp301 | Tyr172, Ala173, Glu174, Gly175, Arg177, Arg262, Thr263, Ala264, Val270, Phe274, Arg277, Phe286, Leu291, Gly295, Thr296, Trp301 |

**Table S6: Predicted Top binders for the SARS-CoV-2 full-length Nucleocapsid Protein POOL predicted Site 3**

| **Ligand** | **ID Number** | **Docking Score (kcal/mol)** | | **MM-GBSA Scores(kcal/mol)** | **Interactions** | **Residues forming the pocket** |
| --- | --- | --- | --- | --- | --- | --- |
| Adenosine-5'-Pentaphosphate | **DB02738** | -19.66 | | -61.40 | **H-Bond:**  Ala156, Leu159, Ala173, Gly175, Arg177, Thr263, Trp301, Ala311  **π- π:**  Trp301 | Thr54, Ala155, Ala156,Val158, Leu159, Gln160, Tyr172, Ala173, Glu174, Gly175, Ser176, Arg177, Lys261, Arg262, Thr263, Thr296, Trp301, Pro302, Ala305, Gln306, Ser310, Ala311, Ser312 |
| Cangrelor | **ZINC000085537017** | -17.52 | -68.96 | | **H-Bond:**  Gly175, Ser176, Arg177, Lys261, Trp301  **π- π:**  Trp301  **Halogen-Bond:**  Ala311 | Thr54, Leu56, Ala156,Val158, Leu159, Gln160, Leu161, Leu167, Tyr172, Ala173, Glu174, Gly175, Ser176, Arg177, Lys261, Thr296, Asp297, Tyr298, Trp301, Pro302, Ala305, Gln306, Ala308, Ser310, Ala311 |
| Etheno-Nadp | **DB03732** | -16.44 | -32.99 | | **H-Bond:**  Ser176, Arg177, Lys261, Thr263, Trp301, Pro309  **π- π:**  Trp301 | Thr54, Ala156, Val158, Leu159, Tyr172, Ala173, Glu174, Gly175, Ser176, Arg177, Gln260, Lys261, Arg262, Thr263, Thr296, Trp301, Pro302, Ala305, Pro309, Ser310, Ala311, Phe314 |
| 6-(adenosine tetraphosphate-methyl)-7,8-dihydropterin | **DB04158** | -14.99 | -57.43 | | **H-Bond:**  Gly175, Ser176, Arg177, Lys261, Trp301  **π- π:**  Trp301 | Thr54, Ala156, Val158, Tyr172, Ala173, Glu174, Gly175, Ser176, Arg177, Gln260, Lys261, Thr263, Thr296, Trp301, Pro302, Ala305, Ala308, Pro309, Ser310, Ala311, Phe314 |
| Adenosine-5'-Rp-Alpha-Thio-Triphosphate | **DB02355** | -14.82 | -43.10 | | **H-Bond:**  Ala173, Arg177, Gln260, Lys261, Arg262, Trp301, Ala311  **π- π:**  Trp301 **π- cation:**  Arg177 | Thr54, Ala155, Ala156, Val158, Ala173, Glu174, Gly175, Ser176, Arg177, Gln260, Lys261, Thr263, Phe274, Trp301, Ala305, Ala308, Pro309, Ser310, Ala311 |
